# Detection in Orchards of Predominant Azole-Resistant *Candida tropicalis* Genotype Causing Human Candidemia, Taiwan

**DOI:** 10.3201/eid3011.240545

**Published:** 2024-11

**Authors:** Kuo-Yun Tseng, Yin-Zhi Chen, Zi-Li Zhou, Jyh-Nong Tsai, Min-Nan Tseng, Hsing-Lung Liu, Chi-Jung Wu, Yu-Chieh Liao, Chih-Chao Lin, De-Jiun Tsai, Feng-Jui Chen, Li-Yun Hsieh, Kuan-Chung Huang, Chun-Hua Huang, Kai-Ting Chen, Wen-Li Chu, Chiao-Mei Lin, Shu-Man Shih, Chao Agnes Hsiung, Yee-Chun Chen, Huey-Kang Sytwu, Yun-Liang Yang, Hsiu-Jung Lo

**Affiliations:** National Tsing Hua University, Hsinchu, Taiwan (K.-Y. Tseng); National Health Research Institutes, Miaoli, Taiwan (K.-Y. Tseng, Y.-Z. Chen, Z.-L. Zhou, C.-J. Wu, Y.-C. Liao, C.-C. Lin, D.-J. Tsai, F.-J. Chen, L.-Y. Hsieh, K.-C. Huang, C.-H. Huang, K.-T. Chen, W.-L. Chu, C.-M. Lin, S.-M. Shih, C.A. Hsiung, Y.-C. Chen, H.-K. Sytwu, H.-J. Lo); National Yang Ming Chiao Tung University, Hsinchu (Z.-L. Zhou, Y.-L. Yang, H.-J. Lo); Taiwan Agricultural Research Institute, Taichung, Taiwan (J.-N. Tsai); Kaohsiung District Agricultural Research and Extension Station, Pingtung, Taiwan (M.-N. Tseng); Taichung District Agricultural Research and Extension Station, CHangHua, Taiwan (H.-L. Liu); National Cheng Kung University Hospital and Medical College, Tainan, Taiwan (C.-J. Wu); National Taiwan University Hospital College of Medicine, Taipei, Taiwan (Y.-C. Chen); China Medical University, Taichung (H.-J. Lo)

**Keywords:** candidemia, *Candida tropicalis*, azole resistance, drug resistance, environment, orchards, pathogenic yeasts, multilocus sequence typing, antimicrobial resistance, fungi, Taiwan

## Abstract

Fluconazole-resistant clade 4 *Candida tropicalis* causing candidemia in humans has been detected in tropical/subtropical areas, including those in China, Singapore, and Australia. We analyzed 704 individual yeasts isolated from fruits, soil, water, and farmers at 80 orchards in Taiwan. The most common pathogenic yeast species among 251 isolates recovered from farmers were *Candida albicans* (14.7%) and *C. parapsilosis* (11.6%). In contrast, *C. tropicalis* (13.0%), *C. palmioleophila* (6.6%), and *Pichia kudriavzevii* (6.0%) were prevalent among 453 environmental isolates. Approximately 18.6% (11/59) of *C. tropicalis* from the environment were resistant to fluconazole, and 81.8% (9/11) of those belonged to the clade 4 genotype. *C. tropicalis* susceptibility to fluconazole correlated with susceptibilities to the agricultural azole fungicides, difenoconazole, tebuconazole, and triadimenol. Tandem gene duplications of mutated *ERG11* contributed to azole resistance. Agriculture environments are a reservoir for azole-resistant *C. tropicalis*; discontinuing agricultural use of azoles might reduce emergence of azole-resistant *Candida* spp. strains in humans.

*Candida* spp. account for ≈8%–15% of invasive infections leading to hospitalization; the emergence of drug-resistant non–*C*. *albicans* *Candida* spp. is particularly troublesome for optimal health recovery of immunocompromised patients, especially those in hospital intensive care units (*1*,*2*). *C. tropicalis* is one of the leading non–*C. albicans* species causing candidemia in humans residing in tropical Asia and Latin America (*3*–*5*) and is also the leading cause of invasive candidiasis in patients with hematologic malignancies (*4*,*6*). Because of major differences in geographic distribution of human-invasive *Candida* spp., each country or region must conduct its own surveillance program to assess the dominant species and emergence of drug-resistant strains (*1*,*2*,*7*). The recent sporadic outbreaks of multidrug-resistant *C. auris* infections in >14 countries located in 5 continents demonstrate the still unmet need for this surveillance (*8*,*9*).

The National Health Research Institutes (NHRI) of Taiwan established the Taiwan Surveillance of Antimicrobial Resistance of Yeasts (TSARY) program in 1999 to periodically monitor national trends in species distribution and antifungal drug susceptibility of pathogenic yeasts isolated from patients (*10*). Subsequent surveillance was conducted in 2002, 2006, 2010, 2014, and 2018 (*11*–*15*). Resistance to fluconazole was found in 25 of 294 *C. tropicalis* isolates from TSARY 2014 and in 31 of 314 *C. tropicalis* isolates from TSARY 2018; moreover, 91.1% (51/56) of fluconazole-resistant *C. tropicalis* belonged to the clade 4 genotype (*15*). Since 2014, other studies have also reported the isolation of azole-resistant *C. tropicalis*, particularly in the Asia-Pacific region (*16*–*18*). Tandem gene duplications of the *ERG11* gene encoding a Y132F amino acid mutation have reportedly contributed to azole-resistant phenotypes in clade 4 *C. tropicalis* isolates from China, Singapore, and Australia (*19*). However, little data have been generated regarding the genetics of *Candida* spp. collected from environmental sources.

*C. tropicalis* has been recovered from soil and aquatic environments (*20*,*21*). Furthermore, the azole-resistant *C. tropicalis* clade 4 genotype has been isolated from fruits purchased at a supermarket in northern Taiwan (*22*). Thus, identifying potential sources of the fluconazole-resistant *C. tropicalis* clade 4 genotype became critical. *Aspergillus fumigatus* fungi recovered from soil and compost have been reported to be genetically related to clinically resistant isolates, raising the possibility that an environmental source of azole-resistant *C. tropicalis* might account for resistant strains in humans (*23*). Through collaborations with healthcare and agriculture sectors, we determined whether the azole-resistant *C. tropicalis* clade 4 genotype existed in orchard environments by investigating the distribution of yeasts in 3 different types of orchards. We also analyzed potential mechanisms contributing to azole resistance in *C. tropicalis*.

## Materials and Methods

### Study Design

We designed this study to evaluate yeast species distribution by using an orchard survey and to investigate the genetic relatedness among fluconazole-resistant *C. tropicalis* isolates. NHRI designed an orchard survey that was used by agricultural research institutes and stations located in representative regions of Taiwan. NHRI’s Human Experiment and Ethics Committee approved the orchard survey (study no. EC1070117).

Most orchards in Taiwan are located in central and southern areas; therefore, we surveyed a total of 80 orchards—31 papaya, 28 wax apple, and 21 grape orchards—located in those 2 areas during July 2012–January 2013. We designated this survey as orchard survey 2012. We collected 1 fruit and 1 soil sample from east, west, south, north, and center collection points within each orchard. Thus, we collected 5 fruit and 5 soil samples from each orchard. From the orchard farmers, we collected swab samples from armpits and hands, as well as oral mouth rinse samples. We analyzed a total of 400 fruit and 400 soil samples, 80 samples of water, and 80 samples each of armpit swab, hand swab, and mouth rinse samples.

### Microbiologic Processing

We isolated yeasts from samples as previously described (*21*,*24*). In brief, we maintained all swab samples at room temperature and transported them to the laboratory within 24 hours after collection. We streaked the samples onto BBL CHROMagar *Candida* plates (BD Biosciences, https://www.bdbiosciences.com). We identified the isolates by rDNA sequencing of the internal transcribed spacer or the D1/D2 domain regions (*25*) and submitted all novel rDNA sequences to GenBank (Appendix Table 1, 2). We further analyzed 1 isolate per species per type of sample. We labeled the strains from orchard survey 2012 with YFA12 and strains from TSARY 2014 with YM14 followed by 4 numbers.

### Drug Susceptibility Testing

Because *Pichia kudriavzevii* (formerly *C. krusei*) is intrinsically resistant to fluconazole, we determined susceptibilities of *C*. *albicans*, *C*. *parapsilosis*, *C*. *tropicalis*, and *Nakaseomyces glabratus* (formerly *C*. *glabrata*), all common yeasts causing human infections, to 2–64 mg/L fluconazole. We analyzed susceptibilities of all 66 *C. tropicalis* isolates to difenoconazole (1–32 mg/L), tebuconazole (1–32 mg/L), and triadimenol (2–64 mg/L), 3 commonly used fungicides in agriculture in Taiwan. We incubated cultures at 35°C for 24 hours in RPMI medium 1640 (Thermo Fisher Scientific, https://www.thermofisher.com) and measured the growth of each isolate by using a Multiskan FC microplate photometer (Thermo Fisher Scientific). We defined MICs as the concentration of drug capable of reducing the turbidity of cells by >50%. We used procedures and clinical breakpoints for yeast strains as previously described (*26*). For fluconazole, the clinical breakpoints for *C. albicans*, *C. parapsilosis*, and *C. tropicalis* were MICs of <2 mg/L for susceptible, >8 mg/L for resistant, and 4 mg/L for susceptible-dose dependent. For *N. glabratus*, we considered a fluconazole MIC of <32 mg/L to be susceptible-dose dependent and >64 mg/L resistant. The breakpoints for fungicides in agriculture have not been defined. We used Spearman correlation coefficient analysis to evaluate the correlations between susceptibilities to fluconazole and to agricultural fungicides. We used the following guide to evaluate the strength of the correlation: very weak, 0.00–0.19; weak, 0.20–0.39; moderate, 0.40–0.59; strong, 0.60–0.79; and very strong, 0.80–1.0.

### Multilocus Sequence Typing

We conducted multilocus sequence typing (MLST) as described previously (*27*,*28*). In brief, we sequenced DNA fragments of 6 *C. tropicalis* genes, *ICL1*, *MDR1*, *SAPT2*, *SAPT4*, *XYR1*, and *ZWF1a*, by using specific primers (Appendix Table 2) and included those sequences in the analyses. We aligned the sequences by using BioNumerics 3.0 (Applied Maths, https://www.applied-maths.com) and compared them with *C. tropicalis* sequences in the public MLST database (http://pubmlst.org) to determine the level of sequence identities and diploid sequence type (DST). We performed phylogenetic analysis by using the unweighted pair group method with arithmetic means algorithm and MEGA 11 software (https://www.megasoftware.net) as previously described (*29*). We determined the genome types of 66 *C. tropicalis* isolates recovered from the orchards and chose a cutoff p-distance value of 0.01 because it separated clades that contained known isolates. We generated a global phylogenetic tree of *C. tropicalis* composed of 1,368 DSTs listed in the *C. tropicalis* MLST database, as previously described (*22*).

### Qualitative Analysis of *CDR1*, *ERG11*, and *MDR1* Transcripts by Real-Time PCR

We determined the expression levels of genes involved in azole resistance in 17 *C. tropicalis* isolates collected from orchards and patients, including 14 fluconazole-resistant (11 clade 4 and 1 each of clades 2, 3, and 8) and 3 fluconazole-susceptible isolates. We harvested the cells after growing them to an optical density of 0.7–0.9 in YPD liquid medium (BD Biosciences) at 30°C for 6 hours. After sequencing *CDR1*, *ERG11*, and *MDR1* gene fragments (Appendix Table 2), we normalized expression levels against *ACT1* in each isolate. Then, we used the mRNA levels in a fluconazole-susceptible isolate, YFA120877 (control strain), recovered from orchard survey 2012, as the denominator for normalization.

### Whole-Genome Sequencing and *ERG11* Copy Number Variant Detection

We conducted whole-genome sequencing (WGS) of 14 orchard-derived *C. tropicalis* isolates that had different genotypes, including 8 isolates resistant and 6 susceptible to fluconazole. We also sequenced 16 isolates from patients in TSARY 2014, including 7 resistant and 9 susceptible to fluconazole, and the *C. tropicalis* strain ATCC750 (American Type Culture Collecton, https://www.atcc.org). We constructed a multiplexing nanopore sequencing library with high molecular weight DNAs by using the Ligation Sequencing Kit and Native Barcoding Expansion Kit (both Oxford Nanopore Technologies, https://www.nanoporetech.com) according to the manufacturer’s instructions. We analyzed a standard 72-hour sequencing script by using MinKNOW software (Oxford Nanopore Technologies), collected raw reads, and then basecalled and demultiplexed by using the standalone application guppy (*30*). After inputting an estimated genome size of 15 Mbp, we obtained long-length and high-quality (coverage ×80; i.e., 1.2 Gbp) sequencing reads by using a customized script, GetFastq.py (*31*). We then filtered and aligned the reads against the *ERG11* gene (GenBank accession no. XM_002550939) by using minimap2 (https://github.com/lh3/minimap2) and a customized script to test for copy number variants. This script separated reads containing *ERG11* sequences into 3 groups: single copy *ERG11* reads, multiple-copy *ERG11* reads, and unsure *ERG11* reads that included partial *ERG11* sequences. For the single and multiple copy groups, we selected a reference read and polished with Medaka version 1.4.3 (https://github.com/nanoporetech/medaka).

### Use of Azole Compounds in Taiwan

Fluconazole (1992), itraconazole (1992), ketoconazole (1981), and voriconazole (2004) have been available in Taiwan for >2 decades. Posaconazole was introduced in 2010. We retrospectively analyzed systemic antifungal drugs administered in healthcare settings in Taiwan by using data obtained from the Taiwan National Health Insurance Research database; the details of the database and methods have been described previously (*32*). We determined the defined daily dose (DDD) of total azole, fluconazole, itraconazole, ketoconazole, and voriconazole in 2005 (1 year after voriconazole was introduced) and 2013 (1 year before TSARY 2014). We did not include the DDD for posaconazole because it was introduced after 2005. We estimated the amounts of azole-type compounds used in agriculture in Taiwan according to Domestic Manufacturers Production & Sale of Pesticides, an annual publication by the Taiwan Crop Protection Industry Association (*33*).

## Results

### Distribution of Yeasts Isolated from Orchard Environment and Farmers

We isolated 704 individual yeasts from 310 samples, including 74 from fruit, 63 from soil, 59 from hand, 58 from oral rinse, 31 from water, and 25 from armpit samples. The isolated yeasts comprised 34 genera and 83 species; 41 of those species have been reported to cause disease in humans. Of the 704 yeasts, 453 (64.3%) were isolated from the environment and 251 (35.7%) from farmers (Table 1; Appendix Table 3). Most (251/453 [55.4%]) environmental isolates were from fruits, and most (126/251 [50.2%]) isolates from farmers were from hand swab samples.

**Table 1:** Distribution of yeasts according to source in study of detection in orchards of predominant azole-resistant *Candida tropicalis* genotype causing human candidemia, Taiwan*

Yeast species (former species name)	Environment					Farmers†				Total
	Fruit	Soil	Water	Subtotal		Armpit	Hand	Oral	Subtotal	
Pathogenic yeasts										
* Candida tropicalis*	18	32	9	59		0	4	3	7	66
* C. albicans*	0	0	2	2		0	2	35	37	39
* C. palmioleophila*	3	26	1	30		1	1	0	2	32
*Pichia kudriavzevii* (*C. krusei*)	11	9	7	27		0	2	2	4	31
* C. parapsilosis*	0	0	0	0		12	12	5	29	29
*Nakaseomyces glabratus* (*C. glabrata*)	0	0	0	0		0	0	3	3	3
* Hanseniaspora opuntiae*	11	3	5	19		1	0	3	4	23
*Moesziomyces aphidis* (*Pseudozyma aphidis*)	11	1	0	12		1	7	2	10	22
*Pichia terricola* (*Issatchenkia terricola*)	9	8	1	18		0	2	1	3	21
* Rhodotorula mucilaginosa*	6	1	0	7		5	9	0	14	21
*Moesziomyces antarcticus* (*Pseudozyma antarctica*)	13	1	0	14		0	5	1	6	20
*Meyerozyma caribbica* (*C. fermentati*)	7	3	4	14		1	1	2	4	18
* Hanseniaspora uvarum*	6	3	0	9		0	3	3	6	15
* Kodamaea ohmeri*	5	1	2	8		0	3	2	5	13
*Meyerozyma guilliermondii* (*C. guilliermondii*)	1	2	0	3		0	1	2	3	6
Other 27 species	13	10	4	27		5	15	7	27	54
Subtotal	114	100	35	249		26	67	71	164	413
Nonpathogenic yeasts										
* Rhodotorula taiwanensis*	26	7	2	35		4	14	3	21	56
*Rhodotorula paludigena* (*Rhodosporidium paludigenum*)	30	8	4	42		0	4	1	5	47
*Sporobolomyces pararoseus* (*Sporidiobolus pararoseus*)	16	3	2	21		0	15	1	16	37
*Rhodosporidiobolus ruineniae* (*Sporidiobolus ruineniae*)	15	5	0	20		0	1	2	3	23
* Hanseniaspora thailandica*	6	5	1	12		0	1	2	3	15
*Papiliotrema aurea* (*Cryptococcus aureus*)	7	0	1	8		0	3	0	3	11
* Pichia occidentalis*	2	7	1	10		0	0	1	1	11
*Starmerella bacillaris* (*C. zemplinina*)	4	0	0	4		0	5	0	5	9
*Rhodotorula toruloides* (*Rhodosporidium toruloides*)	4	0	0	4		0	4	0	4	8
* Debaryomyces nepalensis*	2	3	0	5		1	0	1	2	7
* Pichia manshurica*	3	0	0	3		0	2	2	4	7
*Papiliotrema ruineniae* (*Cryptococcus ruineniae*)	5	0	0	5		0	1	0	1	6
Other 30 species	17	15	3	35		4	9	6	19	54
Subtotal	137	53	14	204		9	59	19	87	291
Total	251	153	49	453		35	126	90	251	704

*Values are numbers of each yeast species isolated from different sources. †Swab samples were collected from hands and armpits of orchard farmers; oral rinse samples were also collected from orchard farmers.

**Figure 1:**
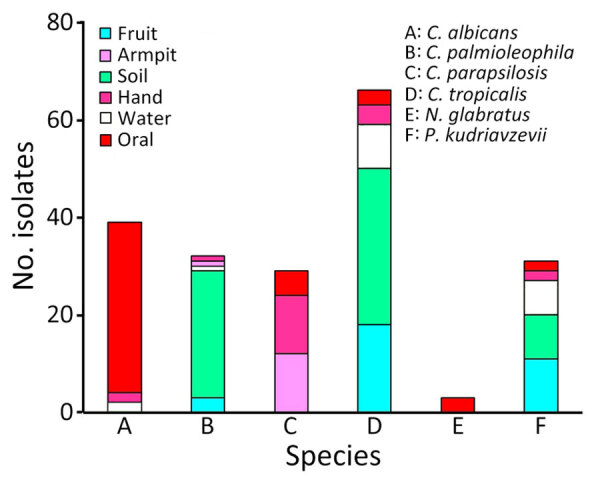
Detection in orchards of predominant azole-resistant *Candida tropicalis* genotype causing human candidemia, Taiwan. Samples from fruit, soil, and water at different orchards were collected. Swab samples from the hands and armpits and oral rinse samples were collected from orchard farmers. Colors indicate source of different yeast species. Numbers of *Candida* spp., *Nakaseomyces glabratus* (formerly *C*. *glabrata*), and *Pichia kudriavzevii* (formerly *C. krusei*) yeast isolates were determined for each sample type.

The most common human pathogenic yeasts recovered from the environment were *C. tropicalis* (59/453 [13.0%]), *Candida palmioleophila* (30/453 [6.6%]), and *P. kudriavzevii* (27/453 [6.0%]), whereas the leading 2 human pathogenic yeasts recovered from the farmers were *C. albicans* (37/251 [14.7%]) and *C. parapsilosis* (29/251 [11.6%]) (Figure 1). Furthermore, yeast species had different prevalences according to the collection site (Table 1). *C. tropicalis* (32/153 [20.9%]) and *C. palmioleophila* (26/153 [17%]) were 2 major species found in soil, and *P. kudriavzevii* was prevalent in water (7/49 [14.3%]). Most (35/37) *C. albicans* and all 3 *N. glabratus* isolated from farmers were found in oral rinse samples. Of the 35 armpit swab samples, 12 (34.3%) contained *C. parapsilosis* and 5 (14.3%) *Rhodotorula mucilaginosa*.

### Susceptibility of 4 Major *Candida* spp. to Fluconazole

All 39 *C. albicans*, 29 *C. parapsilosis*, and 3 *N. glabratus* isolates were susceptible to fluconazole (<2 mg/L), whereas 11 (16.7%) *C. tropicalis* isolates were resistant (>8 mg/L), 14 susceptible-dose dependent (4 mg/L), and 41 susceptible (<2 mg/L) to fluconazole (Table 2). In addition, we found that yeast susceptibilities to fluconazole were positively correlated with susceptibilities to 3 azole fungicides used in agriculture (Table 2). All 11 fluconazole-resistant *C. tropicalis* isolates were collected from the environment in 3 different orchards: grape (n = 4 isolates), papaya (n = 4), and wax apple (n = 3) (Appendix Table 4).

**Table 2:** Correlations between susceptibilities to fluconazole and 3 fungicides in study of detection in orchards of predominant azole-resistant *Candida tropicalis* genotype causing human candidemia, Taiwan*

Fungicide MICs, mg/L	Fluconazole MICs, mg/L						Total no.	Spearman ρ	p value
	<2	4	8	16	32	>64			
Difenoconazole								0.636	<0.001
<1	41	14	1	0	2	0	58		
2	0	0	0	1	2	1	4		
4	0	0	0	0	1	0	1		
>8	0	0	0	0	1	2	3		
Tebuconazole								0.758	<0.001
<1	41	13	0	0	0	0	54		
2	0	1	1	1	5	1	9		
4	0	0	0	0	1	1	2		
>8	0	0	0	0	0	1	1		
Triadimenol								0.875	<0.001
<2	36	1	0	0	0	0	37		
4	1	0	0	0	0	0	1		
8	2	3	1	1	0	0	7		
16	2	9	0	0	0	0	11		
32	0	1	0	0	0	0	1		
>64	0	0	0	0	6	3	9		

*Values are numbers of *C. tropicalis* isolates except as indicated. Spearman correlation coefficients were used to determine correlations between fluconazole susceptibility and susceptibility to 3 different fungicides used in agriculture in Taiwan.

### Genetic Relatedness of *C. tropicalis* Isolates

We analyzed the DSTs of all 66 *C. tropicalis* isolates (Appendix Table 4). The genotype distribution among the 42 fluconazole-susceptible isolates was more diverse, and they were classified into 12 different genotypes: clade 8 (n = 12 isolates); clades 3 and 6 (n = 6 each); clade 4 (n = 5); clade 1 (n = 4); clade 5 (n = 3); clades 2, 10, and 17 (n = 1 each); and DST1394, DST1402, and DST564 (n = 1 each). The 13 fluconazole–susceptible-dose dependent isolates were classified into 5 genotypes: clade 4 (n = 9), clades 8 and 10 (n = 1 each), and DST1394 and DST598 (n = 1 each). Most (≈69%) fluconazole–susceptible-dose dependent isolates belonged to clade 4, of which 88.9% (8/9) belonged to DST225. Furthermore, 9 (81.8%) of 11 fluconazole-resistant isolates belonged to clade 4, consistent with the finding among fluconazole-resistant *C. tropicalis* causing infections in patients.

### Molecular Characteristics of *C. tropicalis* Isolates

We analyzed the expression of genes involved in fluconazole resistance in *C. tropicalis* isolated from orchards and patients (Table 3). *ERG11* mRNA levels increased 3.8–13-fold in all 11 clade 4 isolates compared with that of the azole-susceptible control strain, YFA120877. In contrast, the levels of *ERG11* mRNA in 3 non–clade 4 fluconazole-resistant isolates, including clades 2, 3, and 8, were not increased. In addition to *ERG11*, the expression of *MDR1* in 3 clade 4 fluconazole-resistant and 1 clade 5 fluconazole-susceptible isolates was increased compared with the control. *CDR1* expression in the clade 8 fluconazole-resistant isolate was also increased.

**Table 3:** Characteristics of *Candida tropicalis* isolates from agricultural and clinical settings in study of detection in orchards of predominant azole-resistant *C. tropicalis* genotype causing human candidemia, Taiwan*

Strain	Source	Clade	DST	Fluconazole			Erg11†		Upc2†		mRNA level‡			MLST ID§
				S/R	MIC, mg/L		Y132F/S154F		L168P/A251T/Q287S		*ERG11*	*CDR1*	*MDR1*	
ATCC750	Patient	N914¶	914	S	2		YS/YS		LTS/LTS		ND	ND	ND	1829
YM140066	Ascites	4	506	R	64		YS/FF (9)		LAQ/PTS		5.44	0.64	0.36	831
YM140132	Urine	4	506	R	32		YS/FF (4)		LAQ/LTS		3.08	0.56	0.31	1765
YM140298	Urine	3	585	R	8		YS/YS		LAQ/LAQ		0.67	0	0.26	825
YM140372	Sputum	4	225	R	32		YS/FF (6)		LAQ/LAQ		4.59	0.41	0.24	1762
YM140441	Urine	4	506	R	32		YS/FF (5)		LAQ/PTS		4.88	0.48	0.22	1766
YM140586	Blood	4	225	R	64		YS/FF (9)		LAQ/LTS		4.56	0.57	0.07	833
YM141055	Sputum	2	153	R	>64		YS/YS		LAQ/LAQ		1.24	0.71	1.3	1753
YFA120301	Soil	4	225	R	32		YS/FF (6)		LAQ/PTS		9.59	0.61	2.43	1756
YFA120472	Fruit	8	169	R	16		YS/YS		LAQ/LAQ		1.08	4.24	0.62	1754
YFA120760	Fruit	4	225	R	64		YS/FF (9)		LAQ/PTS		12.98	0.78	2.96	1757
YFA121702	Soil	4	506	R	32		YS/FF (6)		LAQ/PTS		5.81	0.83	2.38	1763
YFA121900-2	Fruit	4	225	R	64		YS/FF (4)		LAQ/PTS		ND	ND	ND	1759
YFA122361	Soil	4	506	R	32		YS/FF (5)		LAQ/LTS		3.81	0.61	0.54	1764
YFA123343-1	Fruit	4	225	R	32		YS/FF (8)		LAQ/PTS		10.85	0.92	0.53	1760
YFA123757	Soil	4	225	R	64		YS/FF (8)		LAQ/PTS		11.28	0.77	1.11	1761
YM140156	Blood	7	139	S	0.25		YS/YS		LAQ/LAQ		ND	ND	ND	1748
YM140225	Blood	5	140	S	0.25		YS/YS		LTS/LTS		ND	ND	ND	1750
YM140458	Sputum	11	923	S	0.25		YS/YS		LAQ/LAS		ND	ND	ND	1767
YM140470	Urine	6	149	S	0.5		YS/YS		LAQ/LAQ		ND	ND	ND	1752
YM140518	Urine	2	134	S	0.25		YS/YS		LAQ/LAQ		ND	ND	ND	1747
YM140896	Blood	5	911	S	0.25		YS/YS		LTS/LTS		1.06	0.57	2.53	843
YM140912	Blood	5	910	S	0.5		YS/YS		LAQ/LAQ		ND	ND	ND	845
YM140977	Blood	5	140	S	0.25		YS/YS		LTS/LTS		ND	ND	ND	1751
YM141031	Urine	4	667	S	0.25		YS/YS		LAQ/LTS		ND	ND	ND	847
YFA120622	Soil	5	928	S	≤2		YS/YS		LAQ/LAQ		ND	ND	ND	818
YFA120679	Hand	5	140	S	≤2		YS/YS		LTS/LTS		ND	ND	ND	1749
YFA120727	Fruit	8	169	S	≤2		YS/YS		LAQ/LAQ		ND	ND	ND	1755
YFA120766	Fruit	1	587	S	≤2		YS/YS		LAQ/LAQ		ND	ND	ND	827
YFA121078	Soil	4	225	S	≤2		YS/FF		LAQ/PTS		1.12	0.8	1.41	1758
YFA121513	Fruit	6	577	S	≤2		YS/YS		LAQ/LAQ		ND	ND	ND	817

*DST, diploid sequence type; ID, identification; MLST, multilocus sequence typing; ND, not determined; R, resistant; S, susceptible. †Locations of amino acid mutations in Erg11 and Upc2 proteins are indicated by numbers. Letter combinations indicate amino acid changes in both alleles of each isolate. Copy numbers of *ERG11* gene are indicated in parentheses, when >1 copy per allele was present. ‡Numbers are fold change in mRNA levels for each gene compared with the control strain (YFA120877). §ID numbers are from the pubMLST database (https://pubmlst.org). ¶Clades were labeled with N followed by DST number if the isolates were not classified into a specific clade.

**Figure 2:**
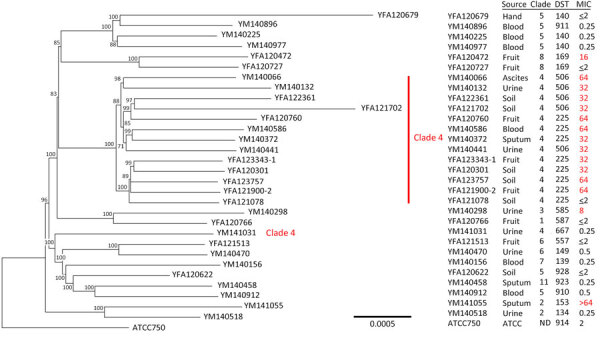
Phylogenetic analysis of *Candida tropicalis* in study of detection in orchards of predominant azole-resistant genotype causing human candidemia, Taiwan. Red numbers indicate fluconazole resistance at MICs of >8 mg/L. We conducted whole-genome sequencing of 31 isolates. Sequence comparisons indicated that all but 1 clade 4 isolate was fluconazole resistant and that isolates from orchards and patients were genetically related. Scale bar indicates nucleotide substitutions per site. ATCC, American Type Tissue Collection; DST, diploid sequence type, ND, not determined.

To further investigate mutations of the azole drug target, *ERG11*, that contributes to fluconazole-resistance we completed WGS of 31 isolates (Table 3). Sequence comparisons indicated that all but 1 clade 4 isolates clustered together (Figure 2), confirming genetic relatedness among fluconazole-resistant isolates from orchards and patients. All 12 clade 4, but not the 3 non–clade 4 (clades 2, 3, and 8), fluconazole-resistant isolates contained Y132F or S154F mutations in the Erg11 protein. In contrast, all but 1 fluconazole-susceptible isolates had wild-type Erg11 protein. The difference between the clade 4 fluconazole-resistant and fluconazole-susceptible isolates was the 4–9 tandem gene duplications of *ERG11* found in resistant isolates, whereas the susceptible isolate, YFA121078, had only 1 copy of the *ERG11* gene.

## Discussion

We found that fluconazole-resistant *C. tropicalis* isolated from fruits, soils, and water in orchards in Taiwan were genetically similar to those causing prolonged colonization and tissues damage in humans. This finding might be associated with both increasingly frequent clinical use of azoles in humans and intense use of azole fungicides in agriculture in Taiwan. We provide epidemiologic evidence indicating that orchards are a reservoir for clade 4 fluconazole-resistant *C. tropicalis*. First, infections in humans were sporadic and unrelated to each other (*15*,*34*), and no identifiable hospital outbreaks occurred. Second, most patients had major underlying conditions and had not been treated with azole drugs within 6 months before hospital admission (*15*). Last, we identified a specific causative clade 4 that shared the same DST genotypes common to both orchards and patients.

Sequential accumulation of adaptive chromosomal mutations has been reported to be associated with drug resistance in fungi (*35*,*36*). Selection of azole-resistant *A. fumigatus* has been associated with agricultural use of azoles (*37*); isolates carrying tandem repeat 34/L98H mutations in the Cyp51A (Erg11) protein were recovered from 2 azole-naive patients with pulmonary aspergillosis (*38*). *C. albicans* and other yeast species isolated from patients with HIV infections and from vine grapes in Bavaria, Germany, were also cross-resistant to medical and agricultural azole drugs (*39*); however, the genetic relatedness among those resistant isolates was not determined.

The YFA121078 isolate was susceptible to fluconazole even though it had both Y132F and S154F mutation in the Erg11 protein, suggesting that >1 mutations in Erg11 might not be sufficient to contribute to treatment failure, but might allow *C. tropicalis* to survive when low levels of azole are present in the environment. The expression of *MDR1* mRNA in YM140896, a fluconazole-susceptible isolate, was 2.5-fold higher than that of the control strain. Furthermore, the expression of *MDR1* mRNA in 3 clade 4 fluconazole-resistant isolates was also higher than that of the control strain. Those findings suggest that overexpression of *MDR1* alone might not cause treatment failure. Our findings are consistent with a concept that fluconazole resistance might be caused by accumulation of molecular changes in different genes, including mutations and overexpression.

An outbreak of *C. auris* in a neuroscience intensive care unit in the United Kingdom was linked to reusable axillary temperature probes (*40*). Among 21 fluconazole-susceptible *C. tropicalis* isolates collected from patients in Italy who had neurologic disorders, 9 belonged to DST747 and 6 to DST333. Isolates from door handles, bedside tables, bed handles, and the hands of healthcare workers also belonged to 1 of those DSTs (*41*). Hence, a specific *Candida* spp. clone can persist in the environment and be horizontally transmitted within a healthcare setting.

The detection of the same clade 4 genotype of fluconazole-resistant *C. tropicalis* from agricultural sites and infected humans suggests that fluconazole-resistant *C. tropicalis* in environments can be a threat to healthcare. A 7-year (2011–2017) observational study of adult patients with *C. tropicalis* bloodstream infections at National Taiwan University Hospital showed that 9 of 58 fluconazole-resistant *C. tropicalis* isolates were DST225 and 6 were DST506, all belonging to clade 4 (*34*). Furthermore, 23 of 30 fluconazole-resistant *C. tropicalis* isolates from Shanghai, China, belonged to clade 4 DST505–7 (*42*). Therefore, active surveillance to detect emergence and dissemination of azole-resistant *C. tropicalis* in clinical settings should be considered and should not be limited to tropical Asia and Latin America.

Because 29 of 31 fluconazole-resistant *C. tropicalis* isolated during TSARY 2018 belonged to the clade 4 genotype, we conducted a follow-up survey of orchards in 2018. Preliminary data showed that >90% of fluconazole-resistant *C. tropicalis* isolates from orchard environments in that survey belonged to clade 4 (H.J. Lo, unpub. data). Our findings demonstrate that the clade 4 fluconazole-resistant *C. tropicalis* genotype is persistent in Taiwan in both clinical settings and the environment.

MLST is a convenient and cost-effective tool to study genetic relatedness and diversity of microbes. We showed that clade assignment from MLST aligned well with the tree topology according to WGS results, which is consistent with our previous report of genetic relatedness among 2 clades detected by MLST and confirmed by mitochondrial genome sequencing (*43*). Nevertheless, when we compared the WGS results among clade 4 strains, we ruled out the possibility that YM141031, which fell outside of the clade 4 cluster (Figure 2), had large insertions or deletions within its genome. Therefore, it is possible that the clade 4 *C. tropicalis* ancestor was divided into 2 different progenies because of drug selection pressure. The fluconazole-susceptible ancestor of YM141031 has wild-type *ERG11* and survives in an azole drug-free environment, whereas the ancestor of YFA121078, found in the clade 4 cluster, has mutations in *ERG11* and survives in the presence of low levels of azole drugs. YFA121078 developed into a fully fluconazole-resistant strain by *ERG11* copy number variation or other potential mechanisms contributing to drug resistance. Thus, the combination of clade 4 genotype and *ERG11* mutations might help to rapidly identify drug-resistant clade 4 *C. tropicalis*.

Twenty-nine different types of azole fungicides have been used in Taiwan. The annual azole fungicide use in agriculture in Taiwan increased from 82.1 tons in 2005 to 145.7 tons in 2013 (*33*). Difenoconazole use increased from 9.2 tons in 2005 to 32 tons in 2013 and tebuconazole increased from 2.1 tons in 2005 to 12.7 tons in 2013; ≈0.5 tons of triadimenol were used during both periods. Correlations between increased agricultural azole use and the appearance of azole resistance in human fungal pathogens have been found. We found that *C. tropicalis* could be traced to a few farmers. However, no farmers harbored clade 4 azole-resistant isolates. Nevertheless, we have established a foundation for more in-depth and systematic studies to evaluate the horizontal transmission of the *C. tropicalis* genotype in the agricultural setting and its implications in the clinical setting.

In conclusion, our findings reemphasize the importance of the One Health concept. In Taiwan, total clinical azole use increased from 14,691 DDD in 2005 to 21,991 DDD in 2013; fluconazole increased from 12,707 DDD in 2005 to 19,053 DDD in 2013, itraconazole increased from 362 DDD in 2005 to 760 DDD in 2013, and voriconazole increased from 332 DDD in 2005 to 1,396 DDD in 2013, whereas ketoconazole decreased from 1,291 DDD in 2005 to 677 DDD in 2013, according to data derived from the Taiwan National Health Insurance Research database (*32*). Antimicrobial drug stewardship efforts in hospitals can reduce the selection of drug-resistant organisms. However, if no efforts are made in agriculture to discontinue use of antimicrobial drug classes used in human medicine, vulnerable patients will continue to become infected with highly resistant organisms and have fewer treatment options. Our findings indicate that agriculture environments are one reservoir for azole-resistant *C. tropicalis*; discontinuing agricultural use of azoles might reduce emergence of azole-resistant *Candida* spp. strains in humans.

Appendix Additional information for detection in orchards of predominant azole-resistant *Candida tropicalis* genotype causing human candidemia, Taiwan.
