# Supplementary material for: Detection in Orchards of Predominant Azole-Resistant Candida tropicalis Genotype Causing Human Candidemia, Taiwan
Source: Emerg Infect Dis. 2024 Nov;30(11):2323–32. doi: 10.3201/eid3011.240545 (PMC11521182; doi:10.3201/eid3011.240545)
Supplement: Appendix — Additional information for detection in orchards of predominant azole-resistant Candida tropicalis genotype causing human candidemia, Taiwan. [file 24-0545-Techapp-s1.pdf]

Article DOI: https://doi.org/10.3201/eid3011.240545

EID cannot ensure accessibility for supplementary materials supplied by authors. Readers who have difficulty accessing supplementary content should contact the authors for assistance.

Detection of Predominant Azole-Resistant *Candida tropicalis* Genotype in Orchards Causing Human Candidemia, Taiwan

Appendix

Appendix Table 1. GenBank accession numbers for novel rDNA sequences of 704 yeast isolates from 80 orchards*

Yeast no.	Farm type	Species	Pathogenic	GenBank accession no.	Novel rDNA sequence (publish date)
YFA121915	Papaya	*Aureobasidium melanogenum*	Y	MG253661	Y (2018 Aug 12)
YFA120052	Grape	*Candida albicans*	Y	KX129762.1	N
YFA120484	Grape	*Candida albicans*	Y	KX129762.1	N
YFA120712	Papaya	*Candida albicans*	Y	KU987839.1	N
YFA120715	Papaya	*Candida albicans*	Y	KU987839.1	N
YFA120742	Papaya	*Candida albicans*	Y	KY101883.1	N
YFA120811	Papaya	*Candida albicans*	Y	KP675457.1	N
YFA121006	Papaya	*Candida albicans*	Y	KU987848.1	N
YFA121024	Papaya	*Candida albicans*	Y	KY101883.1	N
YFA121318	Papaya	*Candida albicans*	Y	MF767826.1	N
YFA121504	Papaya	*Candida albicans*	Y	KX129762.1	N
YFA121543	Papaya	*Candida albicans*	Y	KX129762.1	N
YFA121639	Papaya	*Candida albicans*	Y	KP675539.1	N
YFA121699	Papaya	*Candida albicans*	Y	KY101883.1	N
YFA121816	Papaya	*Candida albicans*	Y	KP675539.1	N
YFA121978	Papaya	*Candida albicans*	Y	KU987848.1	N
YFA122065	Papaya	*Candida albicans*	Y	KU987848.1	N
YFA122080	Papaya	*Candida albicans*	Y	KU987839.1	N
YFA122308	Grape	*Candida albicans*	Y	KY101883.1	N
YFA122452	Grape	*Candida albicans*	Y	KU987848.1	N
YFA122638	Grape	*Candida albicans*	Y	KY101883.1	N
YFA122719	Grape	*Candida albicans*	Y	KM013367.1	N
YFA122869	Wax apple	*Candida albicans*	Y	KX129762.1	N
YFA123265	Wax apple	*Candida albicans*	Y	KY101883.1	N
YFA123268	Wax apple	*Candida albicans*	Y	KX129762.1	N
YFA123337	Wax apple	*Candida albicans*	Y	KY101883.1	N
YFA123574	Wax apple	*Candida albicans*	Y	KY101883.1	N
YFA123853	Wax apple	*Candida albicans*	Y	MF767826.1	N
YFA123904	Wax apple	*Candida albicans*	Y	KU987839.1	N
YFA123922	Wax apple	*Candida albicans*	Y	KX129762.1	N
YFA124147	Wax apple	*Candida albicans*	Y	MF767826.1	N
YFA124333	Wax apple	*Candida albicans*	Y	KY101883.1	N
YFA120637	Papaya	*Candida albicans*	Y	MG253657	Y (2018 Aug 12)
YFA122521	Grape	*Candida albicans*	Y	MG253657	Y (2018 Aug 12)
YFA124012	Wax apple	*Candida albicans*	Y	MG253657	Y (2018 Aug 12)
YFA120772	Papaya	*Candida albicans*	Y	MG253658	Y (2018 Aug 12)
YFA121777	Papaya	*Candida albicans*	Y	MG253659	Y (2018 Aug 12)
YFA121417	Papaya	*Candida albicans*	Y	MG253660	Y (2018 Aug 12)
YFA122731	Grape	*Candida albicans*	Y	MG271801	Y (2018 Aug 22)
YFA123748	Wax apple	*Candida albicans*	Y	MG271802	Y (2018 Aug 22)
YFA122053	Papaya	*Candida duobushaemulonii*	Y	LN864531.1	N
YFA121057	Papaya	*Candida metapsilosis*	Y	KY106577.1	N
YFA121507	Papaya	*Candida metapsilosis*	Y	FM178402.1	N
YFA121510	Papaya	*Candida metapsilosis*	Y	FM178402.1	N
YFA122908	Grape	*Candida metapsilosis*	Y	KY102203.1	N

Yeast no.	Farm type	Species	Pathogenic	GenBank accession no.	Novel rDNA sequence (publish date)
YFA121060	Papaya	*Candida orthopsilosis*	Y	KU665255.1	N
YFA122575	Grape	*Candida orthopsilosis*	Y	KU665255.1	N
YFA122863	Wax apple	*Candida orthopsilosis*	Y	KU665255.1	N
YFA120184	Grape	*Candida palmioleophila*	Y	KU962045.1	N
YFA120376	Grape	*Candida palmioleophila*	Y	KU962045.1	N
YFA120619	Papaya	*Candida palmioleophila*	Y	KU962045.1	N
YFA121123	Papaya	*Candida palmioleophila*	Y	KC479687.1	N
YFA121126	Papaya	*Candida palmioleophila*	Y	KC479687.1	N
YFA121921	Papaya	*Candida palmioleophila*	Y	KC479687.1	N
YFA122503	Grape	*Candida palmioleophila*	Y	KC479687.1	N
YFA122548	Grape	*Candida palmioleophila*	Y	KU962045.1	N
YFA122596	Wax apple	*Candida palmioleophila*	Y	KC479687.1	N
YFA122599	Wax apple	*Candida palmioleophila*	Y	KC479687.1	N
YFA122896	Wax apple	*Candida palmioleophila*	Y	KU962045.1	N
YFA123106	Wax apple	*Candida palmioleophila*	Y	KU962045.1	N
YFA123196	Wax apple	*Candida palmioleophila*	Y	KU962045.1	N
YFA123199	Wax apple	*Candida palmioleophila*	Y	KU962045.1	N
YFA123247	Wax apple	*Candida palmioleophila*	Y	KC479687.1	N
YFA123439	Wax apple	*Candida palmioleophila*	Y	KU962045.1	N
YFA123493	Wax apple	*Candida palmioleophila*	Y	KC479687.1	N
YFA123529	Wax apple	*Candida palmioleophila*	Y	KU962045.1	N
YFA123538	Wax apple	*Candida palmioleophila*	Y	KC479687.1	N
YFA123568	Wax apple	*Candida palmioleophila*	Y	KC479687.1	N
YFA123577	Wax apple	*Candida palmioleophila*	Y	KC479687.1	N
YFA123706	Wax apple	*Candida palmioleophila*	Y	KC479687.1	N
YFA123751	Wax apple	*Candida palmioleophila*	Y	KC479687.1	N
YFA123772	Wax apple	*Candida palmioleophila*	Y	KU962045.1	N
YFA123856	Wax apple	*Candida palmioleophila*	Y	KU962045.1	N
YFA123943	Wax apple	*Candida palmioleophila*	Y	KU962045.1	N
YFA123976	Wax apple	*Candida palmioleophila*	Y	KC479687.1	N
YFA124219	Wax apple	*Candida palmioleophila*	Y	KU962045.1	N
YFA124285	Wax apple	*Candida palmioleophila*	Y	KU962045.1	N
YFA124408	Wax apple	*Candida palmioleophila*	Y	KC479687.1	N
YFA124453	Wax apple	*Candida palmioleophila*	Y	KU962045.1	N
YFA124459	Wax apple	*Candida palmioleophila*	Y	KU962045.1	N
YFA120001	Grape	*Candida parapsilosis*	Y	KX833099.1	N
YFA120004	Grape	*Candida parapsilosis*	Y	KX833099.1	N
YFA120049	Grape	*Candida parapsilosis*	Y	KX833099.1	N
YFA120610	Grape	*Candida parapsilosis*	Y	KX833099.1	N
YFA120670	Papaya	*Candida parapsilosis*	Y	KX833099.1	N
YFA120817	Papaya	*Candida parapsilosis*	Y	KX833099.1	N
YFA120820	Papaya	*Candida parapsilosis*	Y	KX833099.1	N
YFA120832	Papaya	*Candida parapsilosis*	Y	KX833099.1	N
YFA120841	Papaya	*Candida parapsilosis*	Y	KX833099.1	N
YFA120934	Papaya	*Candida parapsilosis*	Y	KX833099.1	N
YFA120943	Papaya	*Candida parapsilosis*	Y	KX833099.1	N
YFA121027	Papaya	*Candida parapsilosis*	Y	KX833099.1	N
YFA121063	Papaya	*Candida parapsilosis*	Y	KX833099.1	N
YFA121069	Papaya	*Candida parapsilosis*	Y	KX833099.1	N
YFA121117	Papaya	*Candida parapsilosis*	Y	KX833099.1	N
YFA121180	Papaya	*Candida parapsilosis*	Y	KX833099.1	N
YFA121546	Papaya	*Candida parapsilosis*	Y	KX833099.1	N
YFA121573	Papaya	*Candida parapsilosis*	Y	KX833099.1	N
YFA121582	Papaya	*Candida parapsilosis*	Y	KX833099.1	N
YFA121837	Papaya	*Candida parapsilosis*	Y	KX833099.1	N
YFA121846	Papaya	*Candida parapsilosis*	Y	KX833099.1	N
YFA121855	Papaya	*Candida parapsilosis*	Y	KX833099.1	N
YFA122323	Grape	*Candida parapsilosis*	Y	KX833099.1	N
YFA122605	Wax apple	*Candida parapsilosis*	Y	KX833099.1	N
YFA122842	Wax apple	*Candida parapsilosis*	Y	KX833099.1	N
YFA122914	Grape	*Candida parapsilosis*	Y	KX833099.1	N
YFA123091	Wax apple	*Candida parapsilosis*	Y	KX833099.1	N
YFA123100	Wax apple	*Candida parapsilosis*	Y	KX833099.1	N
YFA124096	Wax apple	*Candida parapsilosis*	Y	KX833099.1	N
YFA120076	Grape	*Candida tropicalis*	Y	KU950724.1	N
YFA120130	Grape	*Candida tropicalis*	Y	KU950724.1	N
YFA120157	Grape	*Candida tropicalis*	Y	KY711241.1	N
YFA120265	Grape	*Candida tropicalis*	Y	KY711241.1	N

Yeast no.	Farm type	Species	Pathogenic	GenBank accession no.	Novel rDNA sequence (publish date)
YFA120274	Grape	*Candida tropicalis*	Y	KU950724.1	N
YFA120301	Grape	*Candida tropicalis*	Y	KY963120.1	N
YFA120316	Grape	*Candida tropicalis*	Y	KY711241.1	N
YFA120409	Grape	*Candida tropicalis*	Y	KY711241.1	N
YFA120472	Grape	*Candida tropicalis*	Y	KY711241.1	N
YFA120481	Grape	*Candida tropicalis*	Y	KY711241.1	N
YFA120538	Grape	*Candida tropicalis*	Y	KY963120.1	N
YFA120613	Grape	*Candida tropicalis*	Y	KY711241.1	N
YFA120622	Papaya	*Candida tropicalis*	Y	KY963120.1	N
YFA120631	Papaya	*Candida tropicalis*	Y	KY963120.1	N
YFA120664	Papaya	*Candida tropicalis*	Y	KY711241.1	N
YFA120679	Papaya	*Candida tropicalis*	Y	KX129759.1	N
YFA120688	Papaya	*Candida tropicalis*	Y	KY963120.1	N
YFA120718	Papaya	*Candida tropicalis*	Y	KY963120.1	N
YFA120727	Papaya	*Candida tropicalis*	Y	KY963120.1	N
YFA120760	Papaya	*Candida tropicalis*	Y	KY963120.1	N
YFA120767	Papaya	*Candida tropicalis*	Y	KY711241.1	N
YFA120775	Papaya	*Candida tropicalis*	Y	KX129759.1	N
YFA120790	Papaya	*Candida tropicalis*	Y	KY963120.1	N
YFA120853	Papaya	*Candida tropicalis*	Y	KU950724.1	N
YFA120877	Papaya	*Candida tropicalis*	Y	KY711241.1	N
YFA120880	Papaya	*Candida tropicalis*	Y	KU950724.1	N
YFA120925	Papaya	*Candida tropicalis*	Y	KY711241.1	N
YFA120952	Papaya	*Candida tropicalis*	Y	KY963120.1	N
YFA120988	Papaya	*Candida tropicalis*	Y	KY963120.1	N
YFA121078	Papaya	*Candida tropicalis*	Y	KY711241.1	N
YFA121114	Papaya	*Candida tropicalis*	Y	KY711241.1	N
YFA121129	Papaya	*Candida tropicalis*	Y	KY711241.1	N
YFA121135	Papaya	*Candida tropicalis*	Y	KY963120.1	N
YFA121174	Papaya	*Candida tropicalis*	Y	KY963120.1	N
YFA121198	Papaya	*Candida tropicalis*	Y	KY963120.1	N
YFA121240	Papaya	*Candida tropicalis*	Y	KY963120.1	N
YFA121243	Papaya	*Candida tropicalis*	Y	KY711241.1	N
YFA121279	Papaya	*Candida tropicalis*	Y	KY711241.1	N
YFA121315	Papaya	*Candida tropicalis*	Y	KY963120.1	N
YFA121324	Papaya	*Candida tropicalis*	Y	KY711241.1	N
YFA121411	Papaya	*Candida tropicalis*	Y	KU950724.1	N
YFA121513	Papaya	*Candida tropicalis*	Y	KX129759.1	N
YFA121612	Papaya	*Candida tropicalis*	Y	KY963120.1	N
YFA121636	Papaya	*Candida tropicalis*	Y	KX129759.1	N
YFA121642	Papaya	*Candida tropicalis*	Y	KU950724.1	N
YFA121702	Papaya	*Candida tropicalis*	Y	KY711241.1	N
YFA121815	Papaya	*Candida tropicalis*	Y	KY963120.1	N
YFA121825	Papaya	*Candida tropicalis*	Y	KY963120.1	N
YFA121870	Papaya	*Candida tropicalis*	Y	KY963120.1	N
YFA121900-2	Papaya	*Candida tropicalis*	Y	KY711241.1	N
YFA121925	Papaya	*Candida tropicalis*	Y	KY711241.1	N
YFA121990	Papaya	*Candida tropicalis*	Y	KY711241.1	N
YFA122254	Grape	*Candida tropicalis*	Y	KY963120.1	N
YFA122284	Grape	*Candida tropicalis*	Y	KX129759.1	N
YFA122361	Grape	*Candida tropicalis*	Y	KY711241.1	N
YFA122560	Grape	*Candida tropicalis*	Y	KY711241.1	N
YFA122893	Grape	*Candida tropicalis*	Y	KY711241.1	N
YFA122974	Grape	*Candida tropicalis*	Y	KU950724.1	N
YFA123328	Wax apple	*Candida tropicalis*	Y	KY711241.1	N
YFA123343-1	Wax apple	*Candida tropicalis*	Y	KY711241.1	N
YFA123445	Wax apple	*Candida tropicalis*	Y	KY711241.1	N
YFA123611	Wax apple	*Candida tropicalis*	Y	KY711241.1	N
YFA123757	Wax apple	*Candida tropicalis*	Y	KY711241.1	N
YFA123841	Wax apple	*Candida tropicalis*	Y	KY711241.1	N
YFA124159	Wax apple	*Candida tropicalis*	Y	KY711241.1	N
YFA124456	Wax apple	*Candida tropicalis*	Y	KY963120.1	N
YFA121075	Papaya	*Cutaneotrichosporon dermatis (Trichosporon dermatis)*	Y	KT981962.1	N
YFA120874	Papaya	*Cutaneotrichosporon jirovecii (Trichosporon jirovecii)*	Y	HM802131.1	N
YFA120976	Papaya	*Cutaneotrichosporon mucoides (Trichosporon mucoides)*	Y	KX228397.1	N

Yeast no.	Farm type	Species	Pathogenic	GenBank accession no.	Novel rDNA sequence (publish date)
YFA121471	Papaya	*Cystobasidium calyptogenae (Rhodotorula calyptogenae)*	Y	MG211382	Y (2018 Aug 17)
YFA120982	Papaya	*Cystobasidium minutum*	Y	KX349460.1	N
YFA122113	Grape	*Debaryomyces hansenii (Candida famata)*	Y	MG211376.1	N
YFA124441	Wax apple	*Debaryomyces hansenii (Candida famata)*	Y	MG211377.1	N
YFA120736	Papaya	*Diutina catenulata (Candida catenulata)*	Y	KY103373.1	N
YFA120748	Papaya	*Diutina catenulata (Candida catenulata)*	Y	KY103373.1	N
YFA121462	Papaya	*Diutina catenulata (Candida catenulata)*	Y	KY103373.1	N
YFA123241	Wax apple	*Diutina catenulata (Candida catenulata)*	Y	KY103373.1	N
YFA122557	Grape	*Diutina mesorugosa (Candida mesorugosa)*	Y	KY464166.1	N
YFA121021	Papaya	*Diutina rugosa (Candida rugosa)*	Y	MG271804	Y (2018 Aug 22)
YFA122554	Grape	*Diutina rugosa (Candida rugosa)*	Y	MG271804	Y (2018 Aug 22)
YFA122872	Grape	*Diutina rugosa (Candida rugosa)*	Y	MG271804	Y (2018 Aug 22)
YFA120172	Grape	*Hanseniaspora opuntiae*	Y	FM199954.1	N
YFA120196	Grape	*Hanseniaspora opuntiae*	Y	FM199954.1	N
YFA120244	Grape	*Hanseniaspora opuntiae*	Y	FM199954.1	N
YFA120289	Grape	*Hanseniaspora opuntiae*	Y	FM199954.1	N
YFA120307	Grape	*Hanseniaspora opuntiae*	Y	FM199954.1	N
YFA120313	Grape	*Hanseniaspora opuntiae*	Y	FM199954.1	N
YFA120367	Grape	*Hanseniaspora opuntiae*	Y	FM199954.1	N
YFA120457	Grape	*Hanseniaspora opuntiae*	Y	FM199954.1	N
YFA120550	Grape	*Hanseniaspora opuntiae*	Y	FM199954.1	N
YFA120556	Grape	*Hanseniaspora opuntiae*	Y	FM199954.1	N
YFA120592	Grape	*Hanseniaspora opuntiae*	Y	KU316739.1	N
YFA120658	Papaya	*Hanseniaspora opuntiae*	Y	FM199954.1	N
YFA120784	Papaya	*Hanseniaspora opuntiae*	Y	FM199954.1	N
YFA120865	Papaya	*Hanseniaspora opuntiae*	Y	KU316739.1	N
YFA120967	Papaya	*Hanseniaspora opuntiae*	Y	FM199954.1	N
YFA121306	Papaya	*Hanseniaspora opuntiae*	Y	FM199954.1	N
YFA121468	Papaya	*Hanseniaspora opuntiae*	Y	FM199954.1	N
YFA122275	Grape	*Hanseniaspora opuntiae*	Y	KU316739.1	N
YFA122350	Grape	*Hanseniaspora opuntiae*	Y	FM199954.1	N
YFA122455	Grape	*Hanseniaspora opuntiae*	Y	FM199954.1	N
YFA122623	Grape	*Hanseniaspora opuntiae*	Y	FM199954.1	N
YFA122818	Wax apple	*Hanseniaspora opuntiae*	Y	FM199954.1	N
YFA123025	Grape	*Hanseniaspora opuntiae*	Y	FM199954.1	N
YFA122110	Grape	*Hanseniaspora uvarum*	Y	KY816905.1	N
YFA122380	Grape	*Hanseniaspora uvarum*	Y	KT922458.1	N
YFA122419	Grape	*Hanseniaspora uvarum*	Y	KP010405.1	N
YFA122764	Grape	*Hanseniaspora uvarum*	Y	KP010405.1	N
YFA122938	Grape	*Hanseniaspora uvarum*	Y	KP010405.1	N
YFA123073	Grape	*Hanseniaspora uvarum*	Y	KT922458.1	N
YFA123259	Wax apple	*Hanseniaspora uvarum*	Y	KT922458.1	N
YFA123619	Wax apple	*Hanseniaspora uvarum*	Y	KP010405.1	N
YFA123667	Wax apple	*Hanseniaspora uvarum*	Y	KT922458.1	N
YFA123835	Wax apple	*Hanseniaspora uvarum*	Y	KT922458.1	N
YFA124048	Wax apple	*Hanseniaspora uvarum*	Y	KT922458.1	N
YFA124078	Wax apple	*Hanseniaspora uvarum*	Y	KT922458.1	N
YFA124141	Wax apple	*Hanseniaspora uvarum*	Y	KT922458.1	N
YFA122653	Grape	*Hanseniaspora uvarum*	Y	MG251408	Y (2018 Aug 17)
YFA122689	Grape	*Hanseniaspora uvarum*	Y	MG251409	Y (2018 Aug 17)
YFA121768	Papaya	*Hyphopichia burtonii*	Y	KY103598.1	N
YFA120154	Grape	*Kodamaea ohmeri*	Y	JN183447.1	N
YFA120280	Grape	*Kodamaea ohmeri*	Y	KY397951.1	N
YFA120640	Papaya	*Kodamaea ohmeri*	Y	MF599713.1	N
YFA120709	Papaya	*Kodamaea ohmeri*	Y	MF599713.1	N
YFA120814	Papaya	*Kodamaea ohmeri*	Y	MF599713.1	N
YFA120823	Papaya	*Kodamaea ohmeri*	Y	MF599713.1	N
YFA120844	Papaya	*Kodamaea ohmeri*	Y	MF599713.1	N
YFA121465	Papaya	*Kodamaea ohmeri*	Y	MF599713.1	N
YFA121519	Papaya	*Kodamaea ohmeri*	Y	MF599713.1	N

Yeast no.	Farm type	Species	Pathogenic	GenBank accession no.	Novel rDNA sequence (publish date)
YFA121663	Papaya	*Kodamaea ohmeri*	Y	JN183447.1	N
YFA121690	Papaya	*Kodamaea ohmeri*	Y	KY108146.1	N
YFA123262	Wax apple	*Kodamaea ohmeri*	Y	KY397951.1	N
YFA120424	Grape	*Kodamaea ohmeri*	Y	MG211372	Y (2018 Aug 17)
YFA120676	Papaya	*Lodderomyces elongisporus*	Y	FR819714.1	N
YFA121516	Papaya	*Lodderomyces elongisporus*	Y	FR819714.1	N
YFA121567	Papaya	*Lodderomyces elongisporus*	Y	FR819714.1	N
YFA122326	Grape	*Lodderomyces elongisporus*	Y	FR819714.1	N
YFA120022	Grape	*Meyerozyma caribbica (Candida fermentati)*	Y	KY104222.1	N
YFA120109	Grape	*Meyerozyma caribbica (Candida fermentati)*	Y	KY104222.1	N
YFA120163	Grape	*Meyerozyma caribbica (Candida fermentati)*	Y	KY104222.1	N
YFA120217	Grape	*Meyerozyma caribbica (Candida fermentati)*	Y	KY104222.1	N
YFA120259	Grape	*Meyerozyma caribbica (Candida fermentati)*	Y	KY104222.1	N
YFA120439	Grape	*Meyerozyma caribbica (Candida fermentati)*	Y	KY104222.1	N
YFA120478	Grape	*Meyerozyma caribbica (Candida fermentati)*	Y	KY104222.1	N
YFA120496	Grape	*Meyerozyma caribbica (Candida fermentati)*	Y	KY104222.1	N
YFA120547	Grape	*Meyerozyma caribbica (Candida fermentati)*	Y	KY104222.1	N
YFA120589	Grape	*Meyerozyma caribbica (Candida fermentati)*	Y	KY104222.1	N
YFA122191	Grape	*Meyerozyma caribbica (Candida fermentati)*	Y	KY104222.1	N
YFA122962	Grape	*Meyerozyma caribbica (Candida fermentati)*	Y	KY104222.1	N
YFA124084	Wax apple	*Meyerozyma caribbica (Candida fermentati)*	Y	KY104222.1	N
YFA124402	Wax apple	*Meyerozyma caribbica (Candida fermentati)*	Y	KY104222.1	N
YFA123367	Wax apple	*Meyerozyma caribbica (Candida fermentati)*	Y	KY580413	Y (2017 Feb 28)
YFA120919	Papaya	*Meyerozyma caribbica (Candida fermentati)*	Y	KY580413	Y (2017 Feb 28)
YFA123031	Grape	*Meyerozyma caribbica (Candida fermentati)*	Y	KY580413	Y (2017 Feb 28)
YFA123193	Wax apple	*Meyerozyma caribbica (Candida fermentati)*	Y	KY580413	Y (2017 Feb 28)
YFA120364	Grape	*Meyerozyma guilliermondii (Candida guilliermondii)*	Y	KY401420.1	N
YFA121141	Papaya	*Meyerozyma guilliermondii (Candida guilliermondii)*	Y	KY401420.1	N
YFA121579	Papaya	*Meyerozyma guilliermondii (Candida guilliermondii)*	Y	KP675356.1	N
YFA121705	Papaya	*Meyerozyma guilliermondii (Candida guilliermondii)*	Y	KY401420.1	N
YFA124234	Wax apple	*Meyerozyma guilliermondii (Candida guilliermondii)*	Y	KR085964.1	N
YFA124273	Wax apple	*Meyerozyma guilliermondii (Candida guilliermondii)*	Y	JF712904.1	N
YFA124222	Wax apple	*Millerozyma farinosa (Pichia farinosa)*	Y	KY108802.1	N
YFA120826	Papaya	*Moesziomyces antarctica (Pseudozyma antarcticus)*	Y	JN942669.1	N
YFA120826	Papaya	*Moesziomyces antarctica (Pseudozyma antarcticus)*	Y	JN942669.1	N
YFA121213	Papaya	*Moesziomyces antarctica (Pseudozyma antarcticus)*	Y	AB566343.1	N
YFA121552	Papaya	*Moesziomyces antarctica (Pseudozyma antarcticus)*	Y	JN942669.1	N
YFA121564	Papaya	*Moesziomyces antarctica (Pseudozyma antarcticus)*	Y	AB566343.1	N

Yeast no.	Farm type	Species	Pathogenic	GenBank accession no.	Novel rDNA sequence (publish date)
YFA121996	Papaya	*Moesziomyces antarctica (Pseudozyma antarcticus)*	Y	AB566343.1	N
YFA120661	Papaya	*Moesziomyces antarctica (Pseudozyma antarcticus)*	Y	MG211384	Y (2018 Aug 17)
YFA122347	Grape	*Moesziomyces antarctica (Pseudozyma antarcticus)*	Y	AB566343.1	N
YFA120667	Papaya	*Moesziomyces antarctica (Pseudozyma antarcticus)*	Y	MG211384	Y (2018 Aug 17)
YFA120694	Papaya	*Moesziomyces antarctica (Pseudozyma antarcticus)*	Y	MG211384	Y (2018 Aug 17)
YFA120799	Papaya	*Moesziomyces antarctica (Pseudozyma antarcticus)*	Y	MG211384	Y (2018 Aug 17)
YFA121015	Papaya	*Moesziomyces antarctica (Pseudozyma antarcticus)*	Y	MG211384	Y (2018 Aug 17)
YFA121684	Papaya	*Moesziomyces antarctica (Pseudozyma antarcticus)*	Y	MG211384	Y (2018 Aug 17)
YFA121747	Papaya	*Moesziomyces antarctica (Pseudozyma antarcticus)*	Y	MG211384	Y (2018 Aug 17)
YFA122032	Papaya	*Moesziomyces antarctica (Pseudozyma antarcticus)*	Y	MG211384	Y (2018 Aug 17)
YFA122068	Papaya	*Moesziomyces antarctica (Pseudozyma antarcticus)*	Y	MG211384	Y (2018 Aug 17)
YFA122161	Grape	*Moesziomyces antarctica (Pseudozyma antarcticus)*	Y	MG211384	Y (2018 Aug 17)
YFA122200	Grape	*Moesziomyces antarctica (Pseudozyma antarcticus)*	Y	MG211384	Y (2018 Aug 17)
YFA122941	Wax apple	*Moesziomyces antarctica (Pseudozyma antarcticus)*	Y	MG211384	Y (2018 Aug 17)
YFA123007	Grape	*Moesziomyces antarctica (Pseudozyma antarcticus)*	Y	MG211384	Y (2018 Aug 17)
YFA120490	Grape	*Moesziomyces aphidis (Pseudozyma aphidis)*	Y	HQ647298.1	N
YFA120616	Papaya	*Moesziomyces aphidis (Pseudozyma aphidis)*	Y	JN942666.1	N
YFA120655	Papaya	*Moesziomyces aphidis (Pseudozyma aphidis)*	Y	JN942666.1	N
YFA120793	Papaya	*Moesziomyces aphidis (Pseudozyma aphidis)*	Y	JN942666.1	N
YFA120979	Papaya	*Moesziomyces aphidis (Pseudozyma aphidis)*	Y	JN942666.1	N
YFA121036	Papaya	*Moesziomyces aphidis (Pseudozyma aphidis)*	Y	JN942666.1	N
YFA121558	Papaya	*Moesziomyces aphidis (Pseudozyma aphidis)*	Y	HQ647298.1	N
YFA121672	Papaya	*Moesziomyces aphidis (Pseudozyma aphidis)*	Y	HQ647298.1	N
YFA121852	Papaya	*Moesziomyces aphidis (Pseudozyma aphidis)*	Y	HQ647298.1	N
YFA122041	Papaya	*Moesziomyces aphidis (Pseudozyma aphidis)*	Y	JN942666.1	N
YFA122203	Grape	*Moesziomyces aphidis (Pseudozyma aphidis)*	Y	JN942666.1	N
YFA122260	Grape	*Moesziomyces aphidis (Pseudozyma aphidis)*	Y	JN942666.1	N
YFA122581	Grape	*Moesziomyces aphidis (Pseudozyma aphidis)*	Y	HQ647298.1	N
YFA123292	Wax apple	*Moesziomyces aphidis (Pseudozyma aphidis)*	Y	HQ647298.1	N
YFA123400	Wax apple	*Moesziomyces aphidis (Pseudozyma aphidis)*	Y	HQ647298.1	N
YFA123409	Wax apple	*Moesziomyces aphidis (Pseudozyma aphidis)*	Y	HQ647298.1	N
YFA123805	Wax apple	*Moesziomyces aphidis (Pseudozyma aphidis)*	Y	HQ647298.1	N
YFA123877	Wax apple	*Moesziomyces aphidis (Pseudozyma aphidis)*	Y	HQ647298.1	N
YFA124015	Wax apple	*Moesziomyces aphidis (Pseudozyma aphidis)*	Y	HQ647298.1	N

Yeast no.	Farm type	Species	Pathogenic	GenBank accession no.	Novel rDNA sequence (publish date)
YFA121999	Papaya	*Moesziomyces aphidis (Pseudozyma aphidis)*	Y	MG271797	Y (2018 Aug 22)
YFA122722	Grape	*Moesziomyces aphidis (Pseudozyma aphidis)*	Y	MG271797	Y (2018 Aug 22)
YFA122902	Grape	*Moesziomyces aphidis (Pseudozyma aphidis)*	Y	MG271797	Y (2018 Aug 22)
YFA120932	Papaya	*Naganishia liquefaciens (Cryptococcus liquefaciens)*	Y	KM501502.1	N
YFA122317	Grape	*Naganishia liquefaciens (Cryptococcus liquefaciens)*	Y	KM501502.1	N
YFA120634	Papaya	*Nakaseomyces glabratus (Candida glabrata)*	Y	KM103019.1	N
YFA122515	Grape	*Nakaseomyces glabratus (Candida glabrata)*	Y	KM103019.1	N
YFA124330	Wax apple	*Nakaseomyces glabratus (Candida glabrata)*	Y	KU052054.1	N
YFA122716	Grape	*Papiliotrema flavescens (Cryptococcus flavescens)*	Y		N
YFA124126	Wax apple	*Papiliotrema flavescens (Cryptococcus flavescens)*	Y	KY218702.1	N
YFA124444	Wax apple	*Papiliotrema flavescens (Cryptococcus flavescens)*	Y	KY218702.1	N
YFA123550	Wax apple	*Papiliotrema flavescens (Cryptococcus flavescens)*	Y	MG251414	Y (2018 Aug 17)
YFA120142	Grape	*Pichia kudriavzevii (Candida krusei)*	Y	JX174414.1	N
YFA120319	Grape	*Pichia kudriavzevii (Candida krusei)*	Y	JX174414.1	N
YFA120445-1	Grape	*Pichia kudriavzevii (Candida krusei)*	Y	GQ121622.1	N
YFA120451	Grape	*Pichia kudriavzevii (Candida krusei)*	Y	JX174414.1	N
YFA120535	Grape	*Pichia kudriavzevii (Candida krusei)*	Y	KM368825.1	N
YFA120553	Grape	*Pichia kudriavzevii (Candida krusei)*	Y	GQ121622.1	N
YFA120595	Grape	*Pichia kudriavzevii (Candida krusei)*	Y	JX174414.1	N
YFA120604	Grape	*Pichia kudriavzevii (Candida krusei)*	Y	JX174414.1	N
YFA120625	Papaya	*Pichia kudriavzevii (Candida krusei)*	Y	GQ121622.1	N
YFA120721	Papaya	*Pichia kudriavzevii (Candida krusei)*	Y	KX218263.1	N
YFA120739	Papaya	*Pichia kudriavzevii (Candida krusei)*	Y	JX174414.1	N
YFA120745	Papaya	*Pichia kudriavzevii (Candida krusei)*	Y	KX218263.1	N
YFA120787	Papaya	*Pichia kudriavzevii (Candida krusei)*	Y	JX174414.1	N
YFA120805	Papaya	*Pichia kudriavzevii (Candida krusei)*	Y	KX218263.1	N
YFA120847	Papaya	*Pichia kudriavzevii (Candida krusei)*	Y	GQ121622.1	N
YFA120871	Papaya	*Pichia kudriavzevii (Candida krusei)*	Y	GQ121622.1	N
YFA121084	Papaya	*Pichia kudriavzevii (Candida krusei)*	Y	JX174414.1	N
YFA121132	Papaya	*Pichia kudriavzevii (Candida krusei)*	Y	KM368825.1	N
YFA121177	Papaya	*Pichia kudriavzevii (Candida krusei)*	Y	KM368825.1	N
YFA121207	Papaya	*Pichia kudriavzevii (Candida krusei)*	Y	JX174414.1	N
YFA121228	Papaya	*Pichia kudriavzevii (Candida krusei)*	Y	KM368825.1	N
YFA121276	Papaya	*Pichia kudriavzevii (Candida krusei)*	Y	JX174414.1	N
YFA121309	Papaya	*Pichia kudriavzevii (Candida krusei)*	Y	JX174414.1	N
YFA121327	Papaya	*Pichia kudriavzevii (Candida krusei)*	Y	JX174414.1	N
YFA121336	Papaya	*Pichia kudriavzevii (Candida krusei)*	Y	JX174414.1	N
YFA121624	Papaya	*Pichia kudriavzevii (Candida krusei)*	Y	KM368825.1	N
YFA121819	Papaya	*Pichia kudriavzevii (Candida krusei)*	Y	JX174414.1	N
YFA122278	Grape	*Pichia kudriavzevii (Candida krusei)*	Y	JX174414.1	N
YFA122794	Wax apple	*Pichia kudriavzevii (Candida krusei)*	Y	JX174414.1	N
YFA123763	Wax apple	*Pichia kudriavzevii (Candida krusei)*	Y	JX174414.1	N
YFA122506	Grape	*Pichia kudriavzevii (Candida krusei)*	Y	MG815132	Y (2018 Jan 24)
YFA122509	Grape	*Pichia terricola (Issatchenkia terricola)*	Y	MG211383	Y (2018 Aug 17)
YFA120016	Grape	*Pichia terricola (Issatchenkia terricola)*	Y	KY104650.1	N
YFA120190	Grape	*Pichia terricola (Issatchenkia terricola)*	Y	GU237055.1	N
YFA120241	Grape	*Pichia terricola (Issatchenkia terricola)*	Y	GU237055.1	N
YFA120310	Grape	*Pichia terricola (Issatchenkia terricola)*	Y	KY104650.1	N
YFA120322	Grape	*Pichia terricola (Issatchenkia terricola)*	Y	KY104650.1	N

Yeast no.	Farm type	Species	Pathogenic	GenBank accession no.	Novel rDNA sequence (publish date)
YFA120436	Grape	*Pichia terricola (Issatchenkia terricola)*	Y	KY104650.1	N
YFA120505	Grape	*Pichia terricola (Issatchenkia terricola)*	Y	KY104650.1	N
YFA122116	Grape	*Pichia terricola (Issatchenkia terricola)*	Y	KY104650.1	N
YFA122140	Grape	*Pichia terricola (Issatchenkia terricola)*	Y	GU237055.1	N
YFA122188	Grape	*Pichia terricola (Issatchenkia terricola)*	Y	GU237055.1	N
YFA122356	Grape	*Pichia terricola (Issatchenkia terricola)*	Y	GU237055.1	N
YFA122404	Grape	*Pichia terricola (Issatchenkia terricola)*	Y	GU237055.1	N
YFA122482	Grape	*Pichia terricola (Issatchenkia terricola)*	Y	GU237055.1	N
YFA122524	Grape	*Pichia terricola (Issatchenkia terricola)*	Y	GU237055.1	N
YFA122563	Grape	*Pichia terricola (Issatchenkia terricola)*	Y	GU237055.1	N
YFA122617	Grape	*Pichia terricola (Issatchenkia terricola)*	Y	KY104650.1	N
YFA122740	Grape	*Pichia terricola (Issatchenkia terricola)*	Y	KY104650.1	N
YFA122761	Grape	*Pichia terricola (Issatchenkia terricola)*	Y	GU237055.1	N
YFA122815	Wax apple	*Pichia terricola (Issatchenkia terricola)*	Y	GU237055.1	N
YFA122833	Wax apple	*Pichia terricola (Issatchenkia terricola)*	Y	GU237055.1	N
YFA120940	Papaya	*Rhodotorula dairenensis*	Y	KX385849.1	N
YFA124093	Wax apple	*Rhodotorula dairenensis*	Y	KX385849.1	N
YFA121549	Papaya	*Rhodotorula mucilaginosa*	Y	KP223715.1	N
YFA122590	Wax apple	*Rhodotorula mucilaginosa*	Y	LT598656.1	N
YFA122608	Wax apple	*Rhodotorula mucilaginosa*	Y	KY109114.1	N
YFA122911	Grape	*Rhodotorula mucilaginosa*	Y	KP223715.1	N
YFA123076	Wax apple	*Rhodotorula mucilaginosa*	Y	KP223715.1	N
YFA123097	Wax apple	*Rhodotorula mucilaginosa*	Y	KP223715.1	N
YFA123355	Wax apple	*Rhodotorula mucilaginosa*	Y	KP223715.1	N
YFA123427	Wax apple	*Rhodotorula mucilaginosa*	Y	KP223715.1	N
YFA123544	Wax apple	*Rhodotorula mucilaginosa*	Y	KP223715.1	N
YFA123907	Wax apple	*Rhodotorula mucilaginosa*	Y	KP223715.1	N
YFA124033	Wax apple	*Rhodotorula mucilaginosa*	Y	KP223715.1	N
YFA124057	Wax apple	*Rhodotorula mucilaginosa*	Y	KP223715.1	N
YFA124063	Wax apple	*Rhodotorula mucilaginosa*	Y	KP223715.1	N
YFA124075	Wax apple	*Rhodotorula mucilaginosa*	Y	EU781664.1	N
YFA124087	Wax apple	*Rhodotorula mucilaginosa*	Y	KP223715.1	N
YFA124279	Wax apple	*Rhodotorula mucilaginosa*	Y	KP223715.1	N
YFA124294	Wax apple	*Rhodotorula mucilaginosa*	Y	KP223715.1	N
YFA124339	Wax apple	*Rhodotorula mucilaginosa*	Y	KP223715.1	N
YFA122593	Wax apple	*Rhodotorula mucilaginosa*	Y	MG211387	Y (2018 Aug 17)
YFA122881	Grape	*Rhodotorula mucilaginosa*	Y	MG211387	Y (2018 Aug 17)
YFA123736	Wax apple	*Rhodotorula mucilaginosa*	Y	MG211388	Y (2018 Aug 17)
YFA120133	Grape	*Saccharomyces cerevisiae*	Y	KY962551.1	N
YFA120532	Grape	*Saccharomyces cerevisiae*	Y	KY109314.1	N
YFA121822	Papaya	*Saccharomyces cerevisiae*	Y	KT958553.1	N
YFA120082	Grape	*Starmera stellimalicola (Candida stellimalicola)*	Y	MG253655	Y (2018 Aug 12)
YFA120247	Grape	*Starmera stellimalicola (Candida stellimalicola)*	Y	MG253655	Y (2018 Aug 12)
YFA120031	Grape	*Starmera stellimalicola (Candida stellimalicola)*	Y	MG253662	Y (2018 Aug 12)
YFA120427	Grape	*Starmera stellimalicola (Candida stellimalicola)*	Y	MG253662	Y (2018 Aug 12)
YFA120859	Papaya	*Trichosporon asahii*	Y	KY178311.1	N
YFA120937	Papaya	*Trichosporon japonicum*	Y	KY109955.1	N
YFA121072	Papaya	*Trichosporon japonicum*	Y	KY109955.1	N

Yeast no.	Farm type	Species	Pathogenic	GenBank accession no.	Novel rDNA sequence (publish date)
YFA120601	Grape	*Wickerhamomyces anomala (Pichia anomalus)*	Y	KX253664.1	N
YFA120994	Papaya	*Wickerhamomyces anomala (Pichia anomalus)*	Y	KX253664.1	N
YFA121948	Papaya	*Wickerhamomyces anomala (Pichia anomalus)*	Y	KX253664.1	N
YFA123973	Wax apple	*Yarrowia lipolytica (Candida lipolytica)*	Y	KY110196.1	N
YFA124153	Wax apple	*Yarrowia lipolytica (Candida lipolytica)*	Y	KX347557.1	N
YFA124186	Wax apple	*Yarrowia lipolytica (Candida lipolytica)*	Y	KX347557.1	N
YFA123643	Wax apple	*Barnettozyma californica (Zygowilliopsis californica)*	N	KY106172.1	N
YFA121984	Papaya	*Candida akabanensis*	N	KY106274.1	N
YFA123775	Wax apple	*Candida ethanolica*	N	KY102080.1	N
YFA122668	Grape	*Candida incommunis*	N	FM178367.1	N
YFA121861	Papaya	*Candida jiufengensis*	N	MG211375	Y (2018 Aug 17)
YFA120856	Papaya	*Candida sorboxylosa*	N	LC054320.1	N
YFA122422	Grape	*Candida sorboxylosa*	N	LC054320.1	N
YFA120901	Papaya	*Cystobasidium oligophaga (Rhodotorula oligophagum)*	N	KT345341.1	N
YFA121534	Papaya	*Debaryomyces nepalensis*	N	KY103283.1	N
YFA121828	Papaya	*Debaryomyces nepalensis*	N	KY103283.1	N
YFA122167	Grape	*Debaryomyces nepalensis*	N	KY103283.1	N
YFA123103	Wax apple	*Debaryomyces nepalensis*	N	KY103283.1	N
YFA123700	Wax apple	*Debaryomyces nepalensis*	N	KY103283.1	N
YFA124024	Wax apple	*Debaryomyces nepalensis*	N	KY103283.1	N
YFA124429	Wax apple	*Debaryomyces nepalensis*	N	KY103283.1	N
YFA123412	Wax apple	*Hannaella siamensis*	N	AB922850.1	N
YFA120250	Grape	*Hanseniaspora guilliermondii*	N	MG251412	Y (2018 Aug 17)
YFA120385	Grape	*Hanseniaspora guilliermondii*	N	MG251412	Y (2018 Aug 17)
YFA120475	Grape	*Hanseniaspora occidentalis*	N	KY107814.1	N
YFA122134	Grape	*Hanseniaspora occidentalis*	N	KY107814.1	N
YFA122353	Grape	*Hanseniaspora occidentalis*	N	KY107814.1	N
YFA122920	Grape	*Hanseniaspora occidentalis*	N	KY107814.1	N
YFA121741	Papaya	*Hanseniaspora pseudoguilliermondii*	N	KY103547.1	N
YFA121744	Papaya	*Hanseniaspora pseudoguilliermondii*	N	KY103547.1	N
YFA120151	Grape	*Hanseniaspora pseudoguilliermondii*	N	MG251421	Y (2018 Aug 17)
YFA120577	Grape	*Hanseniaspora pseudoguilliermondii*	N	MG251421	Y (2018 Aug 17)
YFA122662	Grape	*Hanseniaspora pseudoguilliermondii*	N	MG493190	Y (2017 Nov 18)
YFA122386	Grape	*Hanseniaspora thailandica*	N	DQ404527.1	N
YFA122518	Grape	*Hanseniaspora thailandica*	N	DQ404527.1	N
YFA122953	Grape	*Hanseniaspora thailandica*	N	DQ404527.1	N
YFA122989	Grape	*Hanseniaspora thailandica*	N	DQ404527.1	N
YFA120034	Grape	*Hanseniaspora thailandica*	N	MG251410	Y (2018 Aug 17)
YFA122734	Grape	*Hanseniaspora thailandica*	N	MG251410	Y (2018 Aug 17)
YFA122020	Papaya	*Hanseniaspora thailandica*	N	MG251411	Y (2018 Aug 17)
YFA122362	Grape	*Hanseniaspora thailandica*	N	MG251411	Y (2018 Aug 17)
YFA122443	Grape	*Hanseniaspora thailandica*	N	MG251411	Y (2018 Aug 17)
YFA122458	Grape	*Hanseniaspora thailandica*	N	MG251411	Y (2018 Aug 17)
YFA122578	Grape	*Hanseniaspora thailandica*	N	MG251411	Y (2018 Aug 17)
YFA122812	Wax apple	*Hanseniaspora thailandica*	N	MG251411	Y (2018 Aug 17)
YFA122923	Grape	*Hanseniaspora thailandica*	N	MG251411	Y (2018 Aug 17)
YFA123214	Wax apple	*Hanseniaspora thailandica*	N	MG251411	Y (2018 Aug 17)
YFA123571	Wax apple	*Hanseniaspora thailandica*	N	MG493194	Y (2017 Nov 18)
YFA120139	Grape	*Hanseniaspora vineae*	N	MG271803	Y (2018 Aug 22)
YFA123325	Wax apple	*Kazachstania humilis (Candida humilis)*	N	KY106507.1	N
YFA121066	Papaya	*Meyerozyma neustonensis (Candida neustonensis)*	N	MG271796	Y (2018 Aug 22)
YFA121282	Papaya	*Meyerozyma neustonensis (Candida neustonensis)*	N	MG271796	Y (2018 Aug 22)
YFA122101	Grape	*Naganishia albidosimilis (Cryptococcus albidosimilis)*	N	LC203701.1	N
YFA121756	Papaya	*Papiliotrema aureus (Cryptococcus aurea)*	N	MG211374	Y (2018 Aug 17)

Yeast no.	Farm type	Species	Pathogenic	GenBank accession no.	Novel rDNA sequence (publish date)
YFA122998	Grape	*Papiliotrema aureus (Cryptococcus aurea)*	N	MG211381	Y (2018 Aug 17)
YFA120574	Grape	*Papiliotrema aureus (Cryptococcus aurea)*	N	DQ640764.1	N
YFA120691	Papaya	*Papiliotrema aureus (Cryptococcus aurea)*	N	DQ640764.1	N
YFA121249	Papaya	*Papiliotrema aureus (Cryptococcus aurea)*	N	DQ640764.1	N
YFA122011	Papaya	*Papiliotrema aureus (Cryptococcus aurea)*	N	HQ832810.1	N
YFA122074	Papaya	*Papiliotrema aureus (Cryptococcus aurea)*	N	HQ832810.1	N
YFA122125	Grape	*Papiliotrema aureus (Cryptococcus aurea)*	N	DQ640764.1	N
YFA122320	Grape	*Papiliotrema aureus (Cryptococcus aurea)*	N	MG251417	Y (2018 Aug 17)
YFA122866	Wax apple	*Papiliotrema aureus (Cryptococcus aurea)*	N	MG251418	Y (2018 Aug 17)
YFA123274	Wax apple	*Papiliotrema aureus (Cryptococcus aurea)*	N	MG251419	Y (2018 Aug 17)
YFA121858	Papaya	*Papiliotrema rajasthanensis (Cryptococcus rajasthanensis)*	N	AM262325.1	N
YFA121972	Papaya	*Papiliotrema ruineniae (Cryptococcus ruineniae)*	N	LK023765.1	N
YFA122935	Grape	*Papiliotrema ruineniae (Cryptococcus ruineniae)*	N	LK023888.1	N
YFA123673	Wax apple	*Papiliotrema ruineniae (Cryptococcus ruineniae)*	N	LK023765.1	N
YFA123730	Wax apple	*Papiliotrema ruineniae (Cryptococcus ruineniae)*	N	LK023765.1	N
YFA123919	Wax apple	*Papiliotrema ruineniae (Cryptococcus ruineniae)*	N	LK023765.1	N
YFA124354	Wax apple	*Papiliotrema ruineniae (Cryptococcus ruineniae)*	N	LK023765.1	N
YFA123622	Wax apple	*Pichia aff. fermentans*	N	HE863822.1	N
YFA123826	Wax apple	*Pichia aff. fermentans*	N	HE863822.1	N
YFA124051	Wax apple	*Pichia aff. fermentans*	N	HE863822.1	N
YFA124432	Wax apple	*Pichia aff. fermentans*	N	HE863822.1	N
YFA121054	Papaya	*Pichia kluyveri*	N	KY580394	N
YFA121159	Papaya	*Pichia kluyveri*	N	KY580396	N
YFA120121	Grape	*Pichia manshurica*	N	KY296081.1	N
YFA120193	Grape	*Pichia manshurica*	N	KY296081.1	N
YFA120286	Grape	*Pichia manshurica*	N	KY296081.1	N
YFA120325	Grape	*Pichia manshurica*	N	KY296081.1	N
YFA120565	Grape	*Pichia manshurica*	N	KY296081.1	N
YFA120607	Grape	*Pichia manshurica*	N	KY296081.1	N
YFA122425	Grape	*Pichia manshurica*	N	KY296081.1	N
YFA120091	Grape	*Pichia occidentalis*	N	KP132530.1	N
YFA120160	Grape	*Pichia occidentalis*	N	KP132530.1	N
YFA121342	Papaya	*Pichia occidentalis*	N	KP132530.1	N
YFA122281	Grape	*Pichia occidentalis*	N	KP132530.1	N
YFA122365	Grape	*Pichia occidentalis*	N	KP132530.1	N
YFA122665	Grape	*Pichia occidentalis*	N	KP132530.1	N
YFA122785	Grape	*Pichia occidentalis*	N	KP132530.1	N
YFA122875	Grape	*Pichia occidentalis*	N	KP132530.1	N
YFA122926	Grape	*Pichia occidentalis*	N	KP132530.1	N
YFA123217	Wax apple	*Pichia occidentalis*	N	KP132530.1	N
YFA123334	Wax apple	*Pichia occidentalis*	N	KP132530.1	N
YFA122272	Grape	*Pichia sporocuriosa*	N	KY104647.1	N
YFA122338	Grape	*Pichia sporocuriosa*	N	KY104647.1	N
YFA122473	Grape	*Pichia sporocuriosa*	N	KY104647.1	N
YFA122671	Grape	*Pichia sporocuriosa*	N	KY104647.1	N
YFA123424	Wax apple	*Pichia sporocuriosa*	N	KY104647.1	N
YFA122551	Grape	*Rhodosporidiobolus ruineniae (Sporidiobolus ruineniae)*	N	FJ743624.1	N
YFA120145	Grape	*Rhodosporidiobolus ruineniae (Sporidiobolus ruineniae)*	N	FJ743624.1	N

Yeast no.	Farm type	Species	Pathogenic	GenBank accession no.	Novel rDNA sequence (publish date)
YFA120238	Grape	*Rhodosporidiobolus ruineniae (Sporidiobolus ruineniae)*	N	FJ743624.1	N
YFA122245	Grape	*Rhodosporidiobolus ruineniae (Sporidiobolus ruineniae)*	N	EU547494.2	N
YFA120013	Grape	*Rhodosporidiobolus ruineniae (Sporidiobolus ruineniae)*	N	MG214668	Y (2018 Aug 17)
YFA120055	Grape	*Rhodosporidiobolus ruineniae (Sporidiobolus ruineniae)*	N	MG214668	Y (2018 Aug 17)
YFA120178	Grape	*Rhodosporidiobolus ruineniae (Sporidiobolus ruineniae)*	N	MG214668	Y (2018 Aug 17)
YFA120220	Grape	*Rhodosporidiobolus ruineniae (Sporidiobolus ruineniae)*	N	MG214668	Y (2018 Aug 17)
YFA120256	Grape	*Rhodosporidiobolus ruineniae (Sporidiobolus ruineniae)*	N	MG214668	Y (2018 Aug 17)
YFA120529	Grape	*Rhodosporidiobolus ruineniae (Sporidiobolus ruineniae)*	N	MG214668	Y (2018 Aug 17)
YFA122155	Grape	*Rhodosporidiobolus ruineniae (Sporidiobolus ruineniae)*	N	MG214668	Y (2018 Aug 17)
YFA122377	Grape	*Rhodosporidiobolus ruineniae (Sporidiobolus ruineniae)*	N	MG214668	Y (2018 Aug 17)
YFA122389	Grape	*Rhodosporidiobolus ruineniae (Sporidiobolus ruineniae)*	N	MG214668	Y (2018 Aug 17)
YFA122437	Grape	*Rhodosporidiobolus ruineniae (Sporidiobolus ruineniae)*	N	MG214668	Y (2018 Aug 17)
YFA122461	Grape	*Rhodosporidiobolus ruineniae (Sporidiobolus ruineniae)*	N	MG214668	Y (2018 Aug 17)
YFA122497	Grape	*Rhodosporidiobolus ruineniae (Sporidiobolus ruineniae)*	N	MG214668	Y (2018 Aug 17)
YFA122710	Grape	*Rhodosporidiobolus ruineniae (Sporidiobolus ruineniae)*	N	MG214668	Y (2018 Aug 17)
YFA122743	Grape	*Rhodosporidiobolus ruineniae (Sporidiobolus ruineniae)*	N	MG214668	Y (2018 Aug 17)
YFA122797	Wax apple	*Rhodosporidiobolus ruineniae (Sporidiobolus ruineniae)*	N	MG214668	Y (2018 Aug 17)
YFA123016	Grape	*Rhodosporidiobolus ruineniae (Sporidiobolus ruineniae)*	N	MG214668	Y (2018 Aug 17)
YFA123187	Wax apple	*Rhodosporidiobolus ruineniae (Sporidiobolus ruineniae)*	N	MG214668	Y (2018 Aug 17)
YFA123520	Wax apple	*Rhodosporidiobolus ruineniae (Sporidiobolus ruineniae)*	N	MG214668	Y (2018 Aug 17)
YFA123940	Wax apple	*Rhodosporidiobolus ruineniae (Sporidiobolus ruineniae)*	N	MG214670	Y (2018 Aug 17)
YFA121216	Papaya	*Rhodotorula paludigenum (Rhodosporidium paludigena)*	N	MG211386	Y (2018 Aug 17)
YFA123175	Wax apple	*Rhodotorula paludigenum (Rhodosporidium paludigena)*	N	MG211386	Y (2018 Aug 17)
YFA120148	Grape	*Rhodotorula paludigenum (Rhodosporidium paludigena)*	N	KY104892.1	N
YFA120205	Grape	*Rhodotorula paludigenum (Rhodosporidium paludigena)*	N	KY104892.1	N
YFA120253	Grape	*Rhodotorula paludigenum (Rhodosporidium paludigena)*	N	KY104892.1	N
YFA120271	Grape	*Rhodotorula paludigenum (Rhodosporidium paludigena)*	N	KY104892.1	N
YFA120571	Grape	*Rhodotorula paludigenum (Rhodosporidium paludigena)*	N	KY104892.1	N
YFA120706	Papaya	*Rhodotorula paludigenum (Rhodosporidium paludigena)*	N	KY104892.1	N
YFA120808	Papaya	*Rhodotorula paludigenum (Rhodosporidium paludigena)*	N	KY104892.1	N
YFA121387	Papaya	*Rhodotorula paludigenum (Rhodosporidium paludigena)*	N	KY104892.1	N
YFA121408	Papaya	*Rhodotorula paludigenum (Rhodosporidium paludigena)*	N	KY104892.1	N
YFA121618	Papaya	*Rhodotorula paludigenum (Rhodosporidium paludigena)*	N	KY104892.1	N
YFA121651	Papaya	*Rhodotorula paludigenum (Rhodosporidium paludigena)*	N	KY104892.1	N

Yeast no.	Farm type	Species	Pathogenic	GenBank accession no.	Novel rDNA sequence (publish date)
YFA121873	Papaya	*Rhodotorula paludigenum (Rhodosporidium paludigena)*	N	KY104892.1	N
YFA121897	Papaya	*Rhodotorula paludigenum (Rhodosporidium paludigena)*	N	KY104892.1	N
YFA122107	Grape	*Rhodotorula paludigenum (Rhodosporidium paludigena)*	N	KY104892.1	N
YFA122137	Grape	*Rhodotorula paludigenum (Rhodosporidium paludigena)*	N	KY104892.1	N
YFA122176	Grape	*Rhodotorula paludigenum (Rhodosporidium paludigena)*	N	KY104892.1	N
YFA122182	Grape	*Rhodotorula paludigenum (Rhodosporidium paludigena)*	N	KY104892.1	N
YFA122197	Grape	*Rhodotorula paludigenum (Rhodosporidium paludigena)*	N	KY104892.1	N
YFA122248	Grape	*Rhodotorula paludigenum (Rhodosporidium paludigena)*	N	KY104892.1	N
YFA122305	Grape	*Rhodotorula paludigenum (Rhodosporidium paludigena)*	N	KY104892.1	N
YFA122383	Grape	*Rhodotorula paludigenum (Rhodosporidium paludigena)*	N	KY104892.1	N
YFA122431	Grape	*Rhodotorula paludigenum (Rhodosporidium paludigena)*	N	KY104892.1	N
YFA122479	Grape	*Rhodotorula paludigenum (Rhodosporidium paludigena)*	N	KY104892.1	N
YFA122512	Grape	*Rhodotorula paludigenum (Rhodosporidium paludigena)*	N	KY104892.1	N
YFA122566	Grape	*Rhodotorula paludigenum (Rhodosporidium paludigena)*	N	KY104892.1	N
YFA122584	Grape	*Rhodotorula paludigenum (Rhodosporidium paludigena)*	N	KT807881.1	N
YFA122626	Grape	*Rhodotorula paludigenum (Rhodosporidium paludigena)*	N	KY104892.1	N
YFA122650	Grape	*Rhodotorula paludigenum (Rhodosporidium paludigena)*	N	KY104892.1	N
YFA122704	Grape	*Rhodotorula paludigenum (Rhodosporidium paludigena)*	N	KY104892.1	N
YFA122767	Grape	*Rhodotorula paludigenum (Rhodosporidium paludigena)*	N	KY104892.1	N
YFA122776	Grape	*Rhodotorula paludigenum (Rhodosporidium paludigena)*	N	KY104892.1	N
YFA122860	Wax apple	*Rhodotorula paludigenum (Rhodosporidium paludigena)*	N	KY104892.1	N
YFA122929	Grape	*Rhodotorula paludigenum (Rhodosporidium paludigena)*	N	KY104892.1	N
YFA122980	Grape	*Rhodotorula paludigenum (Rhodosporidium paludigena)*	N	KY104892.1	N
YFA122995	Grape	*Rhodotorula paludigenum (Rhodosporidium paludigena)*	N	KY104892.1	N
YFA123046	Grape	*Rhodotorula paludigenum (Rhodosporidium paludigena)*	N	KY104892.1	N
YFA123136	Wax apple	*Rhodotorula paludigenum (Rhodosporidium paludigena)*	N	KY104892.1	N
YFA123244	Wax apple	*Rhodotorula paludigenum (Rhodosporidium paludigena)*	N	KY104892.1	N
YFA123256	Wax apple	*Rhodotorula paludigenum (Rhodosporidium paludigena)*	N	KY104892.1	N
YFA123442	Wax apple	*Rhodotorula paludigenum (Rhodosporidium paludigena)*	N	KY104892.1	N
YFA123511	Wax apple	*Rhodotorula paludigenum (Rhodosporidium paludigena)*	N	KY104892.1	N
YFA123676	Wax apple	*Rhodotorula paludigenum (Rhodosporidium paludigena)*	N	KY104892.1	N
YFA123718	Wax apple	*Rhodotorula paludigenum (Rhodosporidium paludigena)*	N	KY104892.1	N
YFA124000	Wax apple	*Rhodotorula paludigenum (Rhodosporidium paludigena)*	N	KY104892.1	N
YFA124372	Wax apple	*Rhodotorula paludigenum (Rhodosporidium paludigena)*	N	KY104892.1	N

Yeast no.	Farm type	Species	Pathogenic	GenBank accession no.	Novel rDNA sequence (publish date)
YFA123514	Wax apple	*Rhodotorula taiwanensis*	N	MG214673	Y (2018 Aug 17)
YFA123910	Wax apple	*Rhodotorula taiwanensis*	N	MG214673	Y (2018 Aug 17)
YFA124270	Wax apple	*Rhodotorula taiwanensis*	N	MG214673	Y (2018 Aug 17)
YFA124336	Wax apple	*Rhodotorula taiwanensis*	N	MG214673	Y (2018 Aug 17)
YFA120802	Papaya	*Rhodotorula taiwanensis*	N	MG211390	Y (2018 Aug 17)
YFA121960	Papaya	*Rhodotorula taiwanensis*	N	MG211390	Y (2018 Aug 17)
YFA122533	Grape	*Rhodotorula taiwanensis*	N	MG211390	Y (2018 Aug 17)
YFA122542	Grape	*Rhodotorula taiwanensis*	N	MG211390	Y (2018 Aug 17)
YFA122611	Wax apple	*Rhodotorula taiwanensis*	N	MG211390	Y (2018 Aug 17)
YFA122821	Wax apple	*Rhodotorula taiwanensis*	N	MG211390	Y (2018 Aug 17)
YFA122830	Wax apple	*Rhodotorula taiwanensis*	N	MG211390	Y (2018 Aug 17)
YFA122884	Grape	*Rhodotorula taiwanensis*	N	MG211390	Y (2018 Aug 17)
YFA123082	Wax apple	*Rhodotorula taiwanensis*	N	MG211390	Y (2018 Aug 17)
YFA123127	Wax apple	*Rhodotorula taiwanensis*	N	MG211390	Y (2018 Aug 17)
YFA123271	Wax apple	*Rhodotorula taiwanensis*	N	MG211390	Y (2018 Aug 17)
YFA123316	Wax apple	*Rhodotorula taiwanensis*	N	MG211390	Y (2018 Aug 17)
YFA123340	Wax apple	*Rhodotorula taiwanensis*	N	MG211390	Y (2018 Aug 17)
YFA123349	Wax apple	*Rhodotorula taiwanensis*	N	MG211390	Y (2018 Aug 17)
YFA123364	Wax apple	*Rhodotorula taiwanensis*	N	MG211390	Y (2018 Aug 17)
YFA123406	Wax apple	*Rhodotorula taiwanensis*	N	MG211390	Y (2018 Aug 17)
YFA123418	Wax apple	*Rhodotorula taiwanensis*	N	MG211390	Y (2018 Aug 17)
YFA123421	Wax apple	*Rhodotorula taiwanensis*	N	MG211390	Y (2018 Aug 17)
YFA123430	Wax apple	*Rhodotorula taiwanensis*	N	MG211390	Y (2018 Aug 17)
YFA123469	Wax apple	*Rhodotorula taiwanensis*	N	MG211390	Y (2018 Aug 17)
YFA123553	Wax apple	*Rhodotorula taiwanensis*	N	MG211390	Y (2018 Aug 17)
YFA123583	Wax apple	*Rhodotorula taiwanensis*	N	MG211390	Y (2018 Aug 17)
YFA123598	Wax apple	*Rhodotorula taiwanensis*	N	MG211390	Y (2018 Aug 17)
YFA123613	Wax apple	*Rhodotorula taiwanensis*	N	MG211390	Y (2018 Aug 17)
YFA123658	Wax apple	*Rhodotorula taiwanensis*	N	MG211390	Y (2018 Aug 17)
YFA123661	Wax apple	*Rhodotorula taiwanensis*	N	MG211390	Y (2018 Aug 17)
YFA123850	Wax apple	*Rhodotorula taiwanensis*	N	MG211390	Y (2018 Aug 17)
YFA123955	Wax apple	*Rhodotorula taiwanensis*	N	MG211390	Y (2018 Aug 17)
YFA123970	Wax apple	*Rhodotorula taiwanensis*	N	MG211390	Y (2018 Aug 17)
YFA124045	Wax apple	*Rhodotorula taiwanensis*	N	MG211390	Y (2018 Aug 17)
YFA124054	Wax apple	*Rhodotorula taiwanensis*	N	MG211390	Y (2018 Aug 17)
YFA124060	Wax apple	*Rhodotorula taiwanensis*	N	MG211390	Y (2018 Aug 17)
YFA124090	Wax apple	*Rhodotorula taiwanensis*	N	MG211390	Y (2018 Aug 17)
YFA124108	Wax apple	*Rhodotorula taiwanensis*	N	MG211390	Y (2018 Aug 17)
YFA124120	Wax apple	*Rhodotorula taiwanensis*	N	MG211390	Y (2018 Aug 17)
YFA124129	Wax apple	*Rhodotorula taiwanensis*	N	MG211390	Y (2018 Aug 17)
YFA124189	Wax apple	*Rhodotorula taiwanensis*	N	MG211390	Y (2018 Aug 17)
YFA124201	Wax apple	*Rhodotorula taiwanensis*	N	MG211390	Y (2018 Aug 17)
YFA124246	Wax apple	*Rhodotorula taiwanensis*	N	MG211390	Y (2018 Aug 17)
YFA124375	Wax apple	*Rhodotorula taiwanensis*	N	MG211390	Y (2018 Aug 17)
YFA124399	Wax apple	*Rhodotorula taiwanensis*	N	MG211390	Y (2018 Aug 17)
YFA123895	Wax apple	*Rhodotorula taiwanensis*	N	MG211391	Y (2018 Aug 17)
YFA121834	Papaya	*Rhodotorula taiwanensis*	N	LC191386.1	N
YFA123280	Wax apple	*Rhodotorula taiwanensis*	N	LC191386.1	N
YFA123283	Wax apple	*Rhodotorula taiwanensis*	N	LC191386.1	N
YFA123286	Wax apple	*Rhodotorula taiwanensis*	N	LC191386.1	N
YFA123799	Wax apple	*Rhodotorula taiwanensis*	N	MG214674	Y (2018 Aug 17)
YFA122848	Wax apple	*Rhodotorula taiwanensis*	N	MG214675	Y (2018 Aug 17)
YFA123682	Wax apple	*Rhodotorula taiwanensis*	N	LC191386.1	N
YFA123724	Wax apple	*Rhodotorula taiwanensis*	N	LC191386.1	N
YFA124009	Wax apple	*Rhodotorula taiwanensis*	N	LC191386.1	N
YFA124312	Wax apple	*Rhodotorula taiwanensis*	N	LC191386.1	N
YFA121273	Papaya	*Rhodotorula toruloides (Rhodosporidium toruloides)*	N	KY109171.1	N
YFA121840	Papaya	*Rhodotorula toruloides (Rhodosporidium toruloides)*	N	MG288675	Y (2018 Sep 26
YFA122104	Grape	*Rhodotorula toruloides (Rhodosporidium toruloides)*	N	MG288675	Y (2018 Sep 26
YFA123088	Wax apple	*Rhodotorula toruloides (Rhodosporidium toruloides)*	N	MG288675	Y (2018 Sep 26
YFA123130	Wax apple	*Rhodotorula toruloides (Rhodosporidium toruloides)*	N	MG288675	Y (2018 Sep 26
YFA123472	Wax apple	*Rhodotorula toruloides (Rhodosporidium toruloides)*	N	MG288675	Y (2018 Sep 26

Yeast no.	Farm type	Species	Pathogenic	GenBank accession no.	Novel rDNA sequence (publish date)
YFA123913	Wax apple	*Rhodotorula toruloides (Rhodosporidium toruloides)*	N	MG288675	Y (2018 Sep 26
YFA123949	Wax apple	*Rhodotorula toruloides (Rhodosporidium toruloides)*	N	MG288675	Y (2018 Sep 26
YFA121537	Papaya	*Rhynchogastrema tunnelae (Bandoniozyma tunnelae)*	N	NR_111074.1	N
YFA122407	Grape	*Saccharomycopsis crataegensis*	N	KR818918.1	N
YFA121192	Papaya	*Saitozyma podzolicus (Cryptococcus podzolica)*	N	KY102922.1	N
YFA120100	Grape	*Sporobolomyces pararoseus (Sporidiobolus pararoseus)*	N	MG214667	Y (2018 Aug 17)
YFA121384	Papaya	*Sporobolomyces pararoseus (Sporidiobolus pararoseus)*	N	MG214667	Y (2018 Aug 17)
YFA121750	Papaya	*Sporobolomyces pararoseus (Sporidiobolus pararoseus)*	N	MG214667	Y (2018 Aug 17)
YFA121888	Papaya	*Sporobolomyces pararoseus (Sporidiobolus pararoseus)*	N	MG214667	Y (2018 Aug 17)
YFA122071	Papaya	*Sporobolomyces pararoseus (Sporidiobolus pararoseus)*	N	MG214667	Y (2018 Aug 17)
YFA122119	Grape	*Sporobolomyces pararoseus (Sporidiobolus pararoseus)*	N	MG214667	Y (2018 Aug 17)
YFA122602	Wax apple	*Sporobolomyces pararoseus (Sporidiobolus pararoseus)*	N	MG214667	Y (2018 Aug 17)
YFA122809	Wax apple	*Sporobolomyces pararoseus (Sporidiobolus pararoseus)*	N	MG214667	Y (2018 Aug 17)
YFA122932	Grape	*Sporobolomyces pararoseus (Sporidiobolus pararoseus)*	N	MG214667	Y (2018 Aug 17)
YFA123295	Wax apple	*Sporobolomyces pararoseus (Sporidiobolus pararoseus)*	N	MG214667	Y (2018 Aug 17)
YFA120103	Grape	*Sporobolomyces pararoseus (Sporidiobolus pararoseus)*	N	KY226609.1	N
YFA120106	Grape	*Sporobolomyces pararoseus (Sporidiobolus pararoseus)*	N	KY226609.1	N
YFA120268	Grape	*Sporobolomyces pararoseus (Sporidiobolus pararoseus)*	N	KY226609.1	N
YFA120433	Grape	*Sporobolomyces pararoseus (Sporidiobolus pararoseus)*	N	KY226609.1	N
YFA120523	Grape	*Sporobolomyces pararoseus (Sporidiobolus pararoseus)*	N	KY226609.1	N
YFA120652	Papaya	*Sporobolomyces pararoseus (Sporidiobolus pararoseus)*	N	KY226609.1	N
YFA121900	Papaya	*Sporobolomyces pararoseus (Sporidiobolus pararoseus)*	N	KY226609.1	N
YFA122128	Grape	*Sporobolomyces pararoseus (Sporidiobolus pararoseus)*	N	KY226609.1	N
YFA122179	Grape	*Sporobolomyces pararoseus (Sporidiobolus pararoseus)*	N	KY226609.1	N
YFA122332	Grape	*Sporobolomyces pararoseus (Sporidiobolus pararoseus)*	N	KY226609.1	N
YFA122344	Grape	*Sporobolomyces pararoseus (Sporidiobolus pararoseus)*	N	KY226609.1	N
YFA122692	Grape	*Sporobolomyces pararoseus (Sporidiobolus pararoseus)*	N	EU003457.1	N
YFA122854	Wax apple	*Sporobolomyces pararoseus (Sporidiobolus pararoseus)*	N	KY226609.1	N
YFA122905	Grape	*Sporobolomyces pararoseus (Sporidiobolus pararoseus)*	N	KY226609.1	N
YFA123004	Grape	*Sporobolomyces pararoseus (Sporidiobolus pararoseus)*	N	KY226609.1	N
YFA123037	Grape	*Sporobolomyces pararoseus (Sporidiobolus pararoseus)*	N	KY226609.1	N
YFA123052	Grape	*Sporobolomyces pararoseus (Sporidiobolus pararoseus)*	N	KY226609.1	N
YFA123085	Wax apple	*Sporobolomyces pararoseus (Sporidiobolus pararoseus)*	N	KY226609.1	N
YFA123277	Wax apple	*Sporobolomyces pararoseus (Sporidiobolus pararoseus)*	N	KY226609.1	N

Yeast no.	Farm type	Species	Pathogenic	GenBank accession no.	Novel rDNA sequence (publish date)
YFA123298	Wax apple	*Sporobolomyces pararoseus (Sporidiobolus pararoseus)*	N	KY226609.1	N
YFA123403	Wax apple	*Sporobolomyces pararoseus (Sporidiobolus pararoseus)*	N	KY226609.1	N
YFA123727	Wax apple	*Sporobolomyces pararoseus (Sporidiobolus pararoseus)*	N	KY226609.1	N
YFA123916	Wax apple	*Sporobolomyces pararoseus (Sporidiobolus pararoseus)*	N	KY226609.1	N
YFA123985	Wax apple	*Sporobolomyces pararoseus (Sporidiobolus pararoseus)*	N	KY226609.1	N
YFA124081	Wax apple	*Sporobolomyces pararoseus (Sporidiobolus pararoseus)*	N	KY226609.1	N
YFA124357	Wax apple	*Sporobolomyces pararoseus (Sporidiobolus pararoseus)*	N	KY226609.1	N
YFA124450	Wax apple	*Sporobolomyces pararoseus (Sporidiobolus pararoseus)*	N	KY226609.1	N
YFA120115	Grape	*Starmerella apicola (Candida apicola)*	N	EU926481.1	N
YFA120118	Grape	*Starmerella bacillaris (Candida zemplinina)*	N	MF574302.1	N
YFA120127	Grape	*Starmerella bacillaris (Candida zemplinina)*	N	MF574302.1	N
YFA121093	Papaya	*Starmerella bacillaris (Candida zemplinina)*	N	MF574302.1	N
YFA122122	Grape	*Starmerella bacillaris (Candida zemplinina)*	N	MF574302.1	N
YFA122335	Grape	*Starmerella bacillaris (Candida zemplinina)*	N	MF574302.1	N
YFA122341	Grape	*Starmerella bacillaris (Candida zemplinina)*	N	MF574302.1	N
YFA122401	Grape	*Starmerella bacillaris (Candida zemplinina)*	N	MF574302.1	N
YFA122944	Grape	*Starmerella bacillaris (Candida zemplinina)*	N	MF574302.1	N
YFA123064	Grape	*Starmerella bacillaris (Candida zemplinina)*	N	MF574302.1	N
YFA121726	Papaya	*Sympodiomycopsis paphiopedili*	N	FJ743630.1	N
YFA120598	Grape	*Torulaspora delbrueckii*	N	MG214671	Y (2018 Aug 17)
YFA120682	Papaya	*Torulaspora delbrueckii*	N	MG214671	Y (2018 Aug 17)
YFA121936	Papaya	*Torulaspora delbrueckii*	N	KP132791.1	N
YFA121333	Papaya	*Torulaspora indica*	N	MG214672	Y (2018 Aug 17)
YFA121366	Papaya	*Torulaspora indica*	N	MG214672	Y (2018 Aug 17)
YFA120961	Papaya	*Trichosporon insectorum*	N	KY109952.1	N
YFA120214	Grape	*Wickerhamiella azyma (Candida azyma)*	N	EF533997.1	N
YFA123436	Wax apple	*Wickerhamomyces pijperi (Pichia pijperi)*	N	MG214676	Y (2018 Aug 17)
YFA123601	Wax apple	*Wickerhamomyces pijperi (Pichia pijperi)*	N	MG214676	Y (2018 Aug 17)
YFA124447	Wax apple	*Wickerhamomyces pijperi (Pichia pijperi)*	N	MG214677	Y (2018 Aug 17)
YFA120199	Grape	*Zygoascus meyerae*	N	KY106016.1	N
YFA122956	Grape	*Zygoascus meyerae*	N	KY106016.1	N

*N, no; Y, yes.

Appendix Table 2. Primers used in study of predominant azole-resistant *Candida tropicalis* genotype in orchards causing human candidemia, Taiwan*

Primer name	Gene	Sequences (5′–3′)	Position†	References
Species identification				
ITS1	rDNA	TCCGTAGGTGAACCTGCGG	NA	(*1*)
ITS4	rDNA	TCCTCCGCTTATTGATATGC	NA	(*1*)
NL1	rDNA	GCATATCAATAAGCGGAGGAAAAG	NA	(*1*)
NL4	rDNA	GGTCCGTGTTTCAAGACGG	NA	(*1*)
Multilocus sequence typing				
HJL2796	*ICL1-F*	CAACAGATTGGTTGCCATCAGAGC	+675 to +698	(*2*,*3*)
HJL2797	*ICL1-R*	CGAAGTCATCAACAGCCAAAGCAG	+1412 to +1435	(*2*,*3*)
HJL2799	*MDR1-F*	GGCTGGAGATGGACTTTTTGG	+772 to +792	(*4*)
HJL2800	*MDR1-R*	TGGAGCACCAAACAATGGG	+1491 to +1509	(*4*)
HJL2801	*SAPT2-F*	CACAGAAACGCCTCCGTTTTG	–46 to –26	(*4*)
HJL2803	*SAPT2-R*	CACTGGTAGCTGAAGGAG	+728 to +745	(*4*)
HJL2804	*SAPT4-F*	TACCCATTGGAATGTATTGTTGA	–103 to –81	(*4*)
HJL2805	*SAPT4-R*	CCAGTTGTTGAATCTGATGAATT	+637 to +659	(*4*)
HJL2807	*XYR1-F*	CCAACAACTATAAAGAGATCGACA	–84 to –61	(*4*)
HJL2808	*XYR1-R*	GCAAGTATGGGTGATGTTCAAT	+580 to +601	(*4*)
HJL2811	*ZWF1a-F*	GTCWTATGATTCMTTYGGWGA	+3 to +23	(*4*)
HJL2812	*ZWF1a-R*	GARACTGGTCTTTCCATRGT	+751 to +770	(*4*)
Real-time PCR				
HJL2780	*ACT1-F*	TGATGGTGTTACCCACGTTGTC	+486 to +507	(*4*)
HJL2781	*ACT1-R*	TCATCAAGTAGTCGGTCAAGTCTCTAC	+563 to +589	(*4*)
HJL2782	*CDR1-F*	AAAATACGGAGCTGATCCATGTC	+3288 to ≈+3310	(*4*)
HJL2783	*CDR1-R*	CGGGTGATTTCATCGTGAACT	+3423 to ≈+3443	(*4*)
HJL2788	*ERG11-F*	GATAAAGGTTTCACCCCAATCAACT	+673 to ≈+697	(*4*)
HJL2789	*ERG11-R*	CCTTCATGTAATGAGCAGATATCTTTCT	+751 to ≈+778	(*4*)
HJL2790	*MDR1-F*	GGAGATGGACTTTTTGGTTTATGTGTAT	+776 to ≈+803	(*4*)
HJL2791	*MDR1-R*	CGACGCAATAAAGTCTTACTGAAAGA	+850 to ≈+875	(*4*)

*NA, not applicable; –, negative; +, positive.
†Position numbers indicate location of gene fragments according to the location of the ATG translation initiation site for each gene, designated as +1.

Appendix Table 3. Distribution of all yeast species isolated in study of predominant azole-resistant *Candida tropicalis* genotype in orchards causing human candidemia, Taiwan*

	Environment				Farmer†				
Yeast species	Fruit	Soil	Water	Subtotal	Armpit	Hand	Oral	Subtotal	Total
Total	251	153	49	453	35	126	90	251	704
Pathogenic yeast									
Aureobasidium melanogenum	0	0	0	0	0	0	1	1	1
Candida albicans	0	0	2	2	0	2	35	37	39
Candida duobushaemulonii	1	0	0	1	0	0	0	0	1
Candida metapsilosis	1	0	0	1	1	2	0	3	4
Candida orthopsilosis	1	0	0	1	0	2	0	2	3
Candida palmioleophila	3	26	1	30	1	1	0	2	32
Candida parapsilosis	0	0	0	0	12	12	5	29	29
Candida tropicalis	18	32	9	59	0	4	3	7	66
Cutaneotrichosporon dermatis (*Trichosporon dermatis*)	0	0	0	0	0	1	0	1	1
Cutaneotrichosporon jirovecii (*Trichosporon jirovecii*)	1	0	0	1	0	0	0	0	1
Cutaneotrichosporon mucoides (*Trichosporon mucoides)*	0	0	0	0	0	0	1	1	1
Cystobasidium minutum	0	0	0	0	0	1	0	1	1
Debaryomyces hansenii (*Candida famata*)	0	1	0	1	0	1	0	1	2
Diutina catenulata (*Candida catenulata*)	0	0	3	3	0	1	0	1	4
Diutina mesorugosa (*Candida mesorugosa*)	0	0	0	0	0	0	1	1	1
Diutina rugosa (*Candida rugosa*)	0	0	1	1	0	0	2	2	3
Hanseniaspora opuntiae	11	3	5	19	1	0	3	4	23
Hanseniaspora uvarum	6	3	0	9	0	3	3	6	15
Hyphopichia burtonii	1	0	0	1	0	0	0	0	1
Kodamaea ohmeri	5	1	2	8	0	3	2	5	13
Lodderomyces elongisporus	1	0	0	1	1	2	0	3	4
Meyerozyma caribbica (*Candida fermentati*)	7	3	4	14	1	1	2	4	18

Yeast species	Environment				Farmer†				Total
	Fruit	Soil	Water	Subtotal	Armpit	Hand	Oral	Subtotal	
Meyerozyma guilliermondii (*Candida guilliermondii*)	1	2	0	3	0	1	2	3	6
Millerozyma farinosa (*Pichia farinosa*)	0	1	0	1	0	0	0	0	1
Moesziomyces antarcticus (*Pseudozyma antarctica*)	13	1	0	14	0	5	1	6	20
Moesziomyces aphidis (*Pseudozyma aphidis*)	11	1	0	12	1	7	2	10	22
Naganishia liquefaciens (*Cryptococcus liquefaciens*)	0	0	0	0	0	2	0	2	2
Nakaseomyces glabratus (*Candida glabrata*)	0	0	0	0	0	0	3	3	3
Papiliotrema flavescens (*Cryptococcus flavescens*)	1	0	0	1	0	0	0	0	1
Papiliotrema laurentii (*Cryptococcus laurentii*)	1	2	0	3	0	0	0	0	3
Pichia kudriavzevii (*Candida krusei*)	11	9	7	27	0	2	2	4	31
Pichia terricola (*Issatchenkia terricola*)	9	8	1	18	0	2	1	3	21
Rhodotorula calyptogenae (*Cystobasidium calyptogenae*)	0	0	0	0	0	1	0	1	1
Rhodotorula dairenensis	0	0	0	0	2	0	0	2	2
Rhodotorula mucilaginosa	6	1	0	7	5	9	0	14	21
Saccharomyces cerevisiae	0	2	0	2	0	1	0	1	3
Starmera stellimalicola (*Candida stellimalicola*)	2	1	0	3	0	0	1	1	4
Trichosporon asahii	1	0	0	1	0	0	0	0	1
Trichosporon japonicum	0	0	0	0	1	1	0	2	2
Wickerhamomyces anomalus (*Pichia anomala*)	2	0	0	2	0	0	1	1	3
Yarrowia lipolytica (*Candida lipolytica*)	0	3	0	3	0	0	0	0	3
Subtotal	114	100	35	249	26	67	71	164	413
Nonpathogenic yeast									
Barnettozyma californica (*Zygowilliopsis californica*)	0	1	0	1	0	0	0	0	1
Candida akabanensis	0	0	0	0	0	0	1	1	1
Candida ethanolica	0	1	0	1	0	0	0	0	1
Candida incommunis	1	0	0	1	0	0	0	0	1
Candida jiufengensis	0	0	0	0	0	1	0	1	1
Candida sorboxylosa	1	0	0	1	0	0	1	1	2
Cystobasidium oligophagum (*Rhodotorula oligophaga*)	0	0	0	0	1	0	0	1	1
Debaryomyces nepalensis	2	3	0	5	1	0	1	2	7
Hannaella siamensis	0	0	0	0	0	1	0	1	1
Hanseniaspora guilliermondii	0	1	0	1	0	0	1	1	2
Hanseniaspora occidentalis	0	2	1	3	1	0	0	1	4
Hanseniaspora pseudoguilliermondii	3	1	1	5	0	0	0	0	5
Hanseniaspora thailandica	6	5	1	12	0	1	2	3	15
Hanseniaspora vineae	0	0	0	0	0	1	0	1	1
Kazachstania humilis (*Candida humilis*)	0	1	0	1	0	0	0	0	1
Meyerozyma neustonensis (*Candida neustonensis)*	0	0	0	0	1	0	1	2	2
Naganishia albidosimilis (*Cryptococcus albidosimilis*)	0	0	0	0	0	1	0	1	1
Papiliotrema aurea (*Cryptococcus aureus*)	7	0	1	8	0	3	0	3	11
Papiliotrema rajasthanensis (*Cryptococcus rajasthanensis*)	0	0	0	0	0	1	0	1	1
Papiliotrema ruineniae (*Cryptococcus ruineniae*)	5	0	0	5	0	1	0	1	6
Pichia aff. fermentans	0	2	0	2	0	1	1	2	4
Pichia kluyveri	1	0	1	2	0	0	0	0	2
Pichia manshurica	3	0	0	3	0	2	2	4	7
Pichia occidentalis	2	7	1	10	0	0	1	1	11
Pichia sporocuriosa	2	1	0	3	1	1	0	2	5
Rhodosporidiobolus ruineniae (*Sporidiobolus ruineniae*)	14	5	0	19	0	1	2	3	22
Rhodotorula paludigena (*Rhodosporidium paludigenum*)	30	8	4	42	0	4	1	5	47

Yeast species	Environment				Farmer†				Total
	Fruit	Soil	Water	Subtotal	Armpit	Hand	Oral	Subtotal	
Rhodotorula taiwanensis	26	7	2	35	4	14	3	21	56
Rhodotorula toruloides (*Rhodosporidium toruloides*)	4	0	0	4	0	4	0	4	8
Rhynchogastrema tunnelae (*Bandoniozyma tunnelae*)	1	0	0	1	0	0	0	0	1
Saccharomycopsis crataegensis	1	0	0	1	0	0	0	0	1
Saitozyma podzolica (*Cryptococcus podzolicus*)	0	1	0	1	0	0	0	0	1
Sporobolomyces pararoseus (*Sporidiobolus pararoseus*)	16	3	2	21	0	15	1	16	37
Starmerella apicola (*Candida apicola*)	0	0	0	0	0	1	0	1	1
Starmerella bacillaris (*Candida zemplinina*)	4	0	0	4	0	5	0	5	9
Sympodiomycopsis paphiopedili	1	0	0	1	0	0	0	0	1
Torulaspora delbrueckii	1	0	0	1	0	1	1	2	3
Torulaspora indica	0	2	0	2	0	0	0	0	2
Trichosporon insectorum	1	0	0	1	0	0	0	0	1
Wickerhamiella azyma (*Candida azyma*)	1	0	0	1	0	0	0	0	1
Wickerhamomyces pijperi (*Pichia pijperi*)	1	2	0	3	0	0	0	0	3
Zygoascus meyerae	2	0	0	2	0	0	0	0	2
Subtotal	137	53	14	204	9	59	19	87	291

*Values are numbers of each yeast species isolated from different sources.

†Swab samples were collected from hands and armpits of orchard farmers; oral rinse samples were also collected from orchard farmers.

Appendix Table 4. Molecular typing of 66 *Candida tropicalis* isolates according to fluconazole susceptibility and source in study of predominant azole-resistant genotype in orchards causing human candidemia, Taiwan*

	Grape orchard						Papaya orchard						Wax apple orchard			
Clade†	Fruit	Soil	Water	Hand	Oral	Subtotal	Fruit	Soil	Water	Hand	Oral	Subtotal	Fruit	Soil	Subtotal	Total
Resistant yeast isolates																
4	0	2	0	0	0	2	3	1	0	0	0	4	1	2	3	9
8	1	1	0	0	0	2	0	0	0	0	0	0	0	0	0	2
Subtotal	1	3	0	0	0	4	3	1	0	0	0	4	1	2	3	11
Susceptible-dose dependent yeast isolates																
4	0	4	0	0	0	4	3	0	1	0	0	4	0	2	2	10
5	0	0	0	0	0	0	0	0	0	0	0	0	0	1	1	1
8	0	0	1	0	0	1	0	0	0	0	0	0	0	0	0	1
10	0	0	0	1	0	1	0	0	0	0	0	0	0	0	0	1
N598	0	1	0	0	0	1	0	0	0	0	0	0	0	0	0	1
Subtotal	0	5	1	1	0	7	3	0	1	0	0	4	0	3	3	14
Susceptible yeast isolates																
1	1	0	0	0	0	1	0	3	0	0	0	3	0	0	0	4
2	0	0	0	0	0	0	0	0	0	0	1	1	0	0	0	1
3	0	0	0	0	0	0	0	2	2	0	1	5	0	1	1	6
4	1	0	0	0	0	1	1	2	0	0	0	3	0	0	0	4
5	0	0	0	0	0	0	0	1	0	1	0	2	0	0	0	2
6	1	0	0	1	0	2	1	0	2	1	0	4	0	0	0	6
8	0	2	0	0	0	2	3	5	2	0	0	10	0	0	0	12
10	0	0	0	0	0	0	0	1	0	0	0	1	0	0	0	1
17	0	0	0	0	0	0	1	0	0	0	0	1	0	0	0	1
N1394	0	0	0	0	0	0	0	1	1	0	0	2	0	0	0	2
N1402	0	0	0	0	0	0	0	0	0	0	0	0	1	0	1	1
N564	0	0	0	0	1	1	0	0	0	0	0	0	0	0	0	1
Subtotal	3	2	0	1	1	7	6	15	7	2	2	32	1	1	2	41
Total	4	10	1	2	1	18	12	16	8	2	2	40	2	6	8	66

**Candida tropicalis* isolates were collected from the environment and from farmers' hands and oral rinses at grape, papaya, and wax apple orchards.
†Clades were labeled with N followed by DST number if the isolates were not classified into a specific clade.

References

1. Leaw SN, Chang HC, Barton R, Bouchara JP, Chang TC. Identification of medically important *Candida* and non-*Candida* yeast species by an oligonucleotide array. J Clin Microbiol. 2007;45:2220–9. PubMed https://doi.org/10.1128/JCM.00543-07

2. Wang SH, Shen M, Lin HC, Sun PL, Lo HJ, Lu JJ. Molecular epidemiology of invasive *Candida albicans* at a tertiary hospital in northern Taiwan from 2003 to 2011. Med Mycol. 2015;53:828–36. PubMed https://doi.org/10.1093/mmy/myv065

3. Chien YS, Chen FJ, Wu HC, Lin CH, Chang WC, Perera D, et al. Cost-effective complete genome sequencing using the MinION platform for identification of recombinant enteroviruses. Microbiol Spectr. 2023;11:e0250723. PubMed https://doi.org/10.1128/spectrum.02507-23

4. Zhou ZL, Tseng KY, Chen YZ, Tsai DJ, Wu CJ, Chen YC, et al. Genetic relatedness among azole-resistant *Candida tropicalis* clinical strains in Taiwan from 2014 to 2018. Int J Antimicrob Agents. 2022;59:106592. PubMed https://doi.org/10.1016/j.ijantimicag.2022.106592
